# Multivariate Correlation Measures Reveal Structure and Strength of Brain–Body Physiological Networks at Rest and During Mental Stress

**DOI:** 10.3389/fnins.2020.602584

**Published:** 2021-02-04

**Authors:** Riccardo Pernice, Yuri Antonacci, Matteo Zanetti, Alessandro Busacca, Daniele Marinazzo, Luca Faes, Giandomenico Nollo

**Affiliations:** ^1^Department of Engineering, University of Palermo, Palermo, Italy; ^2^Department of Physics and Chemistry “Emilio Segrè,” University of Palermo, Palermo, Italy; ^3^Department of Industrial Engineering, University of Trento, Trento, Italy; ^4^Department of Data Analysis, Ghent University, Ghent, Belgium

**Keywords:** network physiology, brain–heart connection, cardiovascular oscillations, EEG waves, physiological stress, time series analysis, wearable devices

## Abstract

In this work, we extend to the multivariate case the classical correlation analysis used in the field of network physiology to probe dynamic interactions between organ systems in the human body. To this end, we define different correlation-based measures of the multivariate interaction (MI) within and between the brain and body subnetworks of the human physiological network, represented, respectively, by the time series of δ, θ, α, and β electroencephalographic (EEG) wave amplitudes, and of heart rate, respiration amplitude, and pulse arrival time (PAT) variability (η, ρ, π). MI is computed: (i) considering all variables in the two subnetworks to evaluate overall brain–body interactions; (ii) focusing on a single target variable and dissecting its global interaction with all other variables into contributions arising from the same subnetwork and from the other subnetwork; and (iii) considering two variables conditioned to all the others to infer the network topology. The framework is applied to the time series measured from the EEG, electrocardiographic (ECG), respiration, and blood volume pulse (BVP) signals recorded synchronously via wearable sensors in a group of healthy subjects monitored at rest and during mental arithmetic and sustained attention tasks. We find that the human physiological network is highly connected, with predominance of the links internal of each subnetwork (mainly η−ρ and δ−θ, θ−α, α−β), but also statistically significant interactions between the two subnetworks (mainly η−β and η−δ). MI values are often spatially heterogeneous across the scalp and are modulated by the physiological state, as indicated by the decrease of cardiorespiratory interactions during sustained attention and by the increase of brain–heart interactions and of brain–brain interactions at the frontal scalp regions during mental arithmetic. These findings illustrate the complex and multi-faceted structure of interactions manifested within and between different physiological systems and subsystems across different levels of mental stress.

## Introduction

Network physiology is a novel research field describing the human organism as an integrated network in which nodes correspond to the organs and edges map organ interactions (Bashan et al., 2012; Bartsch et al., 2015; Ivanov et al., 2016). Since the human physiological network is highly dynamic, the strength of the interactions among organs changes over time across different physiological states as a response to cognitive or homeostatic control mechanisms (e.g.: rest or stress; emotion elicitation; consciousness or unconsciousness; wake, sleep, sleep stages), or due to pathological conditions (Jänig, 2008; Bashan et al., 2012; Waterhouse, 2013; Valenza et al., 2016; Zanetti et al., 2019b). The continuous and dynamic interaction among organs is fundamental for maintaining the individual in good health; a failure in such interaction mechanisms could provoke diseases related to organ dysfunctions or, in the worst case, even the collapse of the whole organism (Ivanov et al., 2016). Therefore, taking into account the human body as a whole and investigating the interactions among multiple organs can provide additional information to that obtained focusing on each physiological system individually (Bartsch et al., 2015). This can now also be easily achieved in non-clinical conditions thanks to the widespread adoption of wearable sensors and systems allowing the non-invasive synchronous acquisition of multiple signals from different physiological districts (Heikenfeld et al., 2018; Jovanov, 2019; Pernice et al., 2019c; Vinciguerra et al., 2019).

Among the variety of organ system interactions, brain–heart interactions play an important role since they underlie the activity of the autonomic nervous system (ANS) and the central nervous system (CNS), which are strictly interconnected through anatomical and functional links and influence each other continuously (Thayer et al., 2012; Beissner et al., 2013; Silvani et al., 2016). Effects of such interactions have also practical importance, as, for instance, cerebral diseases like ischemic stroke and transient ischemic attacks can be due to cardiac arrhythmias such as atrial fibrillation (Marini et al., 2005; Buchwald et al., 2016). On the other hand, the heartbeat dynamics are typically affected by the ANS response to emotional stress, arousal, and physical activity (Dimsdale, 2008; Silvani et al., 2016). In particular, it has been shown that both mental load and physiological stress produce repeatable variations not only in the brain activity (Gevins et al., 1998; Berka et al., 2007; Al-shargie et al., 2018), but also in the dynamic control of the cardiovascular function and heart rate variability (HRV) (Petrowski et al., 2017; Kim et al., 2018; Pernice et al., 2018, 2019a); these effects can be of clinical relevance as they can ultimately increase the risk of heart attacks and stroke (Steptoe and Kivimäki, 2013; Al-Shargie et al., 2016). Moreover, besides the interplay between brain and heart, the network of interactions sub-serving the regulation of the homeostatic function encompasses other physiological rhythms, such as the respiratory drive (Pfurtscheller et al., 2019; Javorka et al., 2020), the cardiovascular and baroreflex functions (Krohova et al., 2019, 2020; Ringwood and Bagnall-Hare, 2020), and other less studied but significant vital signs, e.g., including muscular and ocular activities (Ivanov et al., 2017; Boonstra et al., 2019).

In this context, a main challenge that has emerged in the last years is the development of proper time series analysis techniques capable of suitably quantifying the interactions among different physiological systems starting from the output signals measured from the different organs. The pioneering works in the emerging field of network physiology have used simple cross-correlation measures, showing that they can be a reliable tool to quantify brain–body and brain–brain interactions across different sleep states (Bashan et al., 2012; Lin et al., 2020). In fact, cross-correlation is a well-established tool that has been widely used in many fields of biomedical signal processing, e.g., for assessing the connection between pairs of brain areas in functional magnetic resonance imaging (fMRI) (Cao and Worsley, 1999; Li et al., 2009). Crucially, this approach has also been extended to take into account one or more control variables through the so-called partial correlation (Marrelec et al., 2006; Wang et al., 2016). The latter has been widely employed for the study of brain connectivity, where the coupling between two time series is often assessed removing indirect effects from other multiple series through the use of partial correlation matrices (Marrelec et al., 2006; Oliver et al., 2019). More sophisticated analysis techniques have been proposed for the study of dynamic brain–heart and brain–body interactions, e.g., information-theoretic-based measures able to assess the information produced by each physiological system and transferred to the other connected systems starting from their output time series, which exploit, for example, Granger Causality or penalized regression (we refer the reader to Faes et al., 2014; Duggento et al., 2016; Greco et al., 2019; Zanetti et al., 2019a; Antonacci et al., 2020 for further details) or different approaches like the one calculating the maximal information coefficient (Valenza et al., 2016). However, correlation-based measures have the advantage of being simple, computationally efficient, and usable also for short data sequences. These advantages are highly desirable in the field of network physiology where often only short stationary sequences can be obtained in the challenging analysis conditions where physiological states change transiently with time (Ivanov et al., 2016; Valente et al., 2018). Moreover, the availability of efficient estimators favors their implementation in non-invasive IoT applications using wearable sensors and providing real-time evaluations (Baig et al., 2017; Baker et al., 2017; Pernice et al., 2019c; Vinciguerra et al., 2019).

In the present study, the correlation-based approach to the study of physiological interactions is extended to the multivariate case, providing a formalism and a set of measures for quantifying how blocks of time series are correlated, how the correlation between a “target” time series and multiple “source” series can be dissected into meaningful contributions, and how a multivariate implementation of the concept of partial correlation allows to infer the topology of networks of physiological interactions. Specifically, extending our preliminary analyses carried out in Pernice et al. (2019d), we measure the overall brain–body interactions as the multivariate correlation between the time series representative of the different brain rhythms [δ, θ, α, and β electroencephalographic (EEG) power] and the time series of heart rate, respiratory, and pulse arrival time (PAT) variability. Then, for each target time series from one of the two physiological subnetworks (brain or body), we compute interaction measures explaining how the multivariate correlation between the target and the other series arises from within- and between-subnetwork interactions, or from pairwise interactions. Our analysis is performed in a group of young healthy subjects monitored at rest and during different levels of mental stress, mapping the interaction measures across the scalp EEG electrodes to evidence possible regional effects, and assessing the statistical significance of the proposed measures to reconstruct the topology of brain and body interactions in the different physiological states.

## Materials and Methods

### Hardware Used for Data Acquisition

**FIGURE 1:**
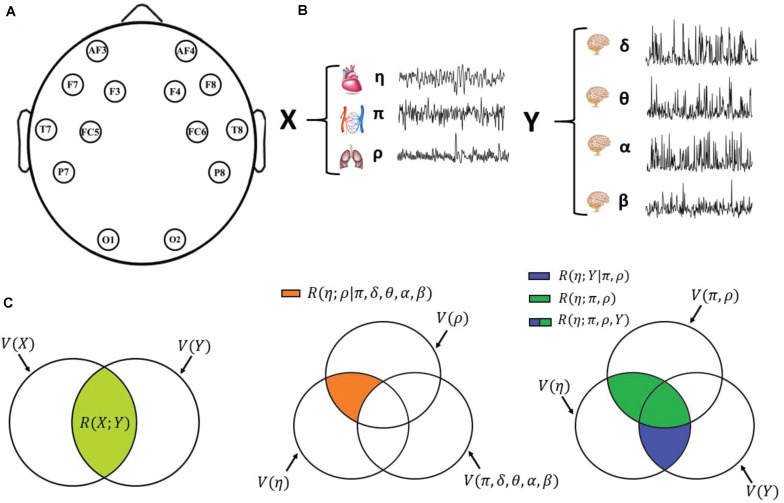
Schematic representation of the data acquisition and analysis steps. **(A)** Graphical representation of the positioning of the 14 EEG electrodes over the scalp. **(B)** Physiological systems and variables considered in this work: cardiac variable η (R–R interval of the ECG), respiratory variable ρ (respiration amplitude), and cardiovascular variable π (pulse arrival time) for the body subnetwork X; amplitude of the δ, θ, α, and β EEG waves for the scalp subnetwork Y; the two subnetworks form the overall physiological network Z. **(C)** Venn diagrams depicting the multivariate interaction measures used in this work: on the left, multivariate brain–body interaction, quantifying the variability shared by the two subnetworks X and Y (light green area); in the middle, “direct” interaction between two individual variables (here, the cardiac and respiratory variables η and ρ), when all other variables are considered (orange area); on the right, decomposition of the interaction between one target variable (here, the cardiac variable η) and all other variables (green + blue areas) as the sum of the interactions internal to the target subnetwork (here, the body subnetwork X; green area) and the interactions exclusive of the other subnetwork (here, the brain subnetwork Y; blue area).

Data used in this study were acquired using non-invasive wearable sensors (Zanetti et al., 2019b). In detail, the signals consisted of electrocardiographic (ECG), EEG, respiratory, and blood volume pulse (BVP) waveforms recorded using different devices. A sensorized t-shirt provided by Smartex (Prato, Italy) was employed for acquiring both the ECG (lead II, sampling frequency of 250 Hz) and the breath signal (sampling rate of 25 Hz). The E4 wristband provided by Empatica (Milano, Italy) with a photoplethysmographic (PPG) sensor has been used for BVP signal (sampling rate of 64 Hz). Finally, for EEG data, the EPOC PLUS wireless headset provided by Emotiv (San Francisco, CA, United States) has been employed, recording 14 signals from electrodes positioned on the scalp according to a reduced version of the 10-20 international placement system (see Figure 1A). All the data were acquired synchronously and sent wirelessly via Bluetooth to a personal computer for the subsequent post-processing and analyses. Particular care has been paid to ensure the correct positioning of the wearable devices on the body. Moreover, an appositely designed method for ensuring synchronization of the different acquired biosignals has been employed, based on the linear warping of the time axis with respect to the Smartex signal taken as a reference. We refer the reader to Zanetti et al. (2019b) for further details and the complete synchronization procedure.

### Measurement Protocol

Eighteen young healthy volunteers (13 males, five females; age range: 20–30 years) were monitored during a measurement protocol consisting of three experimental conditions corresponding to different levels of mental stress (Zanetti et al., 2019a):

(i) A resting condition (REST), lasting 12 min and consisting in watching a video showing landscapes with relaxing background music;
(ii) A sustained attention task (GAME) lasting 12 min and consisting in playing a serious game, i.e., following a cursor on the screen while trying to avoid some obstacles;
(iii) A mental arithmetic test (MENTAL) lasting 7 min during which the volunteer had to carry out the maximum possible number of three-digit sums and subtractions.

The three above-described conditions actually correspond to an increasing level of stress, since a sustained attention task produces higher mental involvement than a fully relaxed state, still not being as stressful as carrying out fast and continuous arithmetic calculations (Zanetti et al., 2019a).

The experiment was approved by the Ethics Committees of the University of Trento. All volunteers participating in this study provided written informed consent. Further details on the measurement protocol employed for this study can be found in Zanetti et al. (2019a).

### Time Series Extraction

Data processing was carried out offline employing MATLAB R2019b (MathWorks, Natick, MA, United States). To allow the analysis of brain–body interactions, the acquired physiological signals were processed extracting synchronous time series representative of the dynamical activity of the body and brain intended as separate physiological districts (sub-networks). ECG recordings were first preprocessed to correct for artifacts and to remove baseline wander and high-frequency noise, respectively, using a high-pass filter (half power frequency of 1 Hz) and a low-pass filter (half power frequency of 20 Hz); zero-phase filtering was adopted to avoid group delays. Afterward, a template matching algorithm (Dobbs et al., 1984; Speranza et al., 1993; Oweis and Al-Tabbaa, 2014; Zanetti et al., 2019a) was employed to extract the R peaks and thus obtain R-R interval (RRI) time series (variable η). R peaks detection was carried out finding the local maxima of the cross-correlation between a template of the QRS complex and the ECG, applying a threshold on the cross-correlation, and finally locating the time of the R peak at the time of the maximum value of the aligned template (Dobbs et al., 1984; Oweis and Al-Tabbaa, 2014). Tachograms were visually inspected to assess the accurate detection of R peaks, or otherwise to correct for missing and ectopic beats (Zanetti et al., 2019a). The breathing signal was sampled at the same time instants of the R peaks in the ECG to obtain the respiratory time series (variable ρ). To assess the dynamical activity of the cardiovascular system, the PAT time series (variable π) was extracted as the sequence of consecutive time intervals between the ECG R peak and the maximum derivative of the BVP signal for each given cardiac cycle (Gao et al., 2016). As regards the brain district, the power spectral density (PSD) was calculated for each EEG signal using a 2-s sliding window with 50% overlap. In each window, the spectral power in the frequency bands 0.5–3, 3–8, 8–12, and 12–25 Hz (respectively, δ, θ, α, and β) was measured through integration of the spectral profile within each band, extracting brain time series which resulted sampled at 1 Hz. The procedure was repeated for the signals recorded from all electrodes, to extract the spatial distribution of the EEG band-power time series. Maps were generated through interpolation, over a 100 × 100 grid, of the values of EEG band-power time series using the MATLAB built-in biharmonic spline method. The interpolation was used only for visualization purposes, while all the analyses were carried out on the acquired data. The brain time series extracted in this way were synchronous with those obtained resampling at 1 Hz the three cardiovascular time series using spline interpolation (Zanetti et al., 2019a). The rate of 1 Hz, which sets a time scale for the analysis which is compatible with the spectrum of heart rhythms, has already been used in previous studies in the field of network physiology for analyzing the time series from different body locations (Bashan et al., 2012; Bartsch et al., 2015). The uniformity of the final sampling rate and the synchronization of the signals acquired from the different devices, carried out according to the procedure described in Section “Hardware Used for Data Acquisition,” permitted to obtain synchronous time series for all the physiological districts. Each time series consisted of 300 samples (corresponding to 5 min of signal recording) and particular care was taken to avoid transient phenomena during the different conditions. This has been accomplished starting the considered time window 3 min after the beginning of the REST phase, and from 1 to 2 min after the start of a MENTAL or GAME condition (not more to avoid habituation of the volunteer to the more stressful condition). All time series were checked for a restricted form of weak sense stationarity using the algorithm proposed in Magagnin et al. (2011), which randomly extracts a given number of sub-windows from each time series and assesses the steadiness of mean and variance across the sub-windows.

In the following, we will denote *X* as the body subnetwork, consisting of the η, ρ, and π variables, while *Y* denotes the brain subnetwork (scalp areas), consisting of the δ, θ, α, and β variables. We are aware that recent studies have highlighted that particular care should be assumed making inferences about brain regions when using EEG signals acquired on the scalp (Lai et al., 2018; Van de Steen et al., 2019), and will discuss this issue in Section “Discussion.” Figure 1 schematically depicts the approach followed in this study, with the time series analyzed (Figure 1B) and the measures of multivariate interaction (MI) (Figure 1C) which are presented in detail in the next subsection.

### Multivariate Interaction Analysis

In this work, the time series measuring the output values of the different physiological systems introduced in the previous section are interpreted as consecutive observations of random variables mapping the system states. A typical approach used in network physiology to study the interactions between two physiological variables *x* and *y* is to quantify their linear correlation (Bashan et al., 2012; Lin et al., 2020). The most common measure is the squared Pearson’s correlation coefficient, defined as:

(1) $$\rho^{2}\,\left(x;y\right)\equiv \frac{\Sigma_{x;y}^{2}}{\Sigma_{x}\,\Sigma_{y}}$$

where Σ_*x*_ = *𝔼*[^(*x*−*m*_*x*_)2^] and Σ_*y*_ = *𝔼*[^(*y*−*m*_*y*_)2^] are the variance of *x* and *y* being *m*_*x*_ = *𝔼*[*x*] and *m*_*y*_ = *𝔼*[*y*] their mean values, and Σ_*x*;*y*_ = *𝔼*[(*x*−*m*_*x*_)(*y*−*m*_*y*_)] is their covariance (*𝔼* represents the expectation operator). The squared correlation is a symmetric normalized measure of linear dependence between *x* and *y*, i.e., ρ^2^(*x*;*y*) = ρ^2^(*y*;*x*), which ranges from 0 to 1 moving from the absence of correlation to full correlation.

While Eq. 1 is the most commonly known expression for the squared correlation, it can also be formulated in terms of the determinant of the covariance matrix of the vector variable concatenating *x* and *y*, *W* = [*xy*], as

$$\rho^{2}\,\left(x;y\right)=1-\frac{\left|\Sigma_{\left[x\,y\right]}\right|}{\Sigma_{x}\,\Sigma_{y}},$$

(2) $$\Sigma_{\left[x\,y\right]}=\Sigma_{W}=𝔼\,\left[{\left(W-m_{W}\right)}^{T}\,\left(W-m_{W}\right)\right],$$

or in terms of the residuals of a simple linear regression model of the type *x*=*ay* + *b* + *u*, where *a* and *b* are the regression coefficients and *u* is the prediction error, as

(3) $$\rho^{2}\,\left(x;y\right)=1-\frac{\Sigma_{x|y}}{\Sigma_{x}},\Sigma_{x|y}=\Sigma_{u},$$

in which Σ_*x*|*y*_ is the so-called partial variance, i.e., the variance of the error of the regression of *x* on *y*. The derivation of Eqs 2 and 3 is reported in the Appendix.

In the present work, we extend the above measures to the multivariate case, considering the random vectors *X* and *Y* that collect the variables of the so-called body subnetwork composed by the cardiac, respiratory, and cardiovascular processes, and the variables of the brain subnetwork composed by the EEG power-band processes. With the notation introduced above, the body and brain variables are the *P*-dimensional vector *X* = [ηρπ] and the *Q*-dimensional vector *Y* = [δθαβ] (*P* = 3,*Q* = 4), which are further grouped in the vector describing the state of the whole physiological network, *Z* = [*XY*]=[*Z*_1_⋯*Z*_*M*_] (*M* = *P* + *Q* = 7). Then, denoting as Σ_*X*_ = [*cpsbreak*]*𝔼*[^(*X*−*m*_*X*_)*T*^(*X*−*m*_*X*_)], Σ_*Y*_ = *𝔼*[^(*Y*−*m*_*Y*_)*T*^(*Y*−*m*_*Y*_)] and Σ_*Z*_ = *𝔼*[^(*Z*−*m*_*Z*_)*T*^(*Z*−*m*_*Z*_)] the *P* × *P*, *Q* × *Q*, and *M* × *M* covariance matrices of *X*, *Y*, and *Z*, we define the *multivariate correlation* between *X* and *Y* extending Eq. 2 as follows:

(4) $$\rho^{2}\,\left(X;Y\right)\equiv 1-\frac{\left|\Sigma_{\left[X\,Y\right]}\right|}{\left|\Sigma_{X}\right|\,\left|\Sigma_{Y}\right|},\Sigma_{\left[X\,Y\right]}=\Sigma_{Z}.$$

This definition also has a straightforward interpretation in terms of linear regression. Indeed, considering the regression *X* = *YA* + *B* + *U*, where *A* and *B* are parameter vectors of dimension *Q* × *P* and 1 × *P*, and *U* is an 1 × *P* vector of residuals, and defining the so-called partial covariance of *X* given *Y* as $\Sigma_{X|Y}\equiv \Sigma_{X}-\Sigma_{X;Y}\,\Sigma_{Y}^{-1}\,\Sigma_{Y;X}$ being Σ_*X*;*Y*_ and Σ_*Y*;*X*_ the cross-covariance matrices (Barnett et al., 2009), it can be shown (see Appendix) that the multivariate correlation can be formulated in analogy to Eq. 3 as

(5) $$\rho^{2}\,\left(X;Y\right)=1-\frac{\left|\Sigma_{X|Y}\right|}{\left|\Sigma_{X}\right|},\Sigma_{X|Y}=\Sigma_{U}.$$

From Eq. 5, it is clear that the squared multivariate correlation is related to the covariance matrix of the prediction error of a multivariate linear regression. Moreover, it is symmetric (ρ^2^(*X*;*Y*) = ρ^2^(*Y*;*X*)) and ranges from 0 to 1, indicating, respectively, uncorrelation (obtained when *A* = 0) and full linear dependence (obtained when *U* = 0) between *X* and *Y*. Here, we further define a logarithmic version of the multivariate correlation between *X* and *Y*, which we denote as MI:

(6) $$R\,\left(X;Y\right)\equiv -\ln\left(1-\rho^{2}\,\left(X;Y\right)\right)=\ln\frac{\left|\Sigma_{X}\right|}{\left|\Sigma_{X|Y}\right|}.$$

The MI measure defined in Eq. 6 is null when *X* and *Y* are uncorrelated and, contrary to the squared correlation, it tends to infinity when *X* and *Y* are completely correlated. Also, we note that the MI can be expressed as the difference between two terms related to the covariance structure of the vector variables as:

(7) R(X;Y)=V(X)-V(X|Y),

where *V*(*X*) = *ln*⁡|Σ_*X*_| is a logarithmic form of the so-called generalized variance of *X* and *V*(*X*|*Y*) = *ln*|Σ_*X*|*Y*_| is the logarithmic generalized partial variance of *X* given *Y* (Barrett et al., 2010), quantifying, respectively, the overall variability within *X* and the part of such variability that remains after regressing *X* on *Y*. Eq. 7 is depicted graphically in the Venn diagram of Figure 1C (left). The MI measure defined in Eqs 6 and 7 is motivated by its link to information-theoretic quantities when the variables are jointly Gaussian (see Appendix), and because it offers the possibility to decompose in a meaningful way the variability shared between group of variables, as seen in the following.

Next, to quantify how a single physiological process is linked to the others, we derive measures of the MI between a scalar variable and a vector variable. To this end, let us consider a “target” scalar variable in the body subnetwork, *x*_*i*_ ∈ *X*, and denote as ^*Xi*^=*X*\*x*_*i*_ the remaining variables in *X* (*i* = 1,…,*P*); similarly, a target variable *y*_*j*_ ∈ *Y* can be chosen in the brain subnetwork, separating it from the other variables ^*Yj*^ = *Y*\*y*_*j*_ (*j* = 1,…,*Q*). Then, the interaction between the target variable and all other variables in the network is defined as:

$$R\,\left(x_{i};X^{i},Y\right)=V\,\left(x_{i}\right)-V\,\left(x_{i}|X^{i},Y\right),$$

(8) $$R\left(y_{j};Y^{j},X\right)=V\left(y_{j}\right)-V\left(y_{j}|Y^{j},X\right),$$

where the generalized variances and partial variances are *V*(*x*_*i*_) = *ln*⁡_Σ*x*_*i*__, *V*(*y*_*j*_) = *ln*⁡_Σ*y*_*j*__, and *V*(*x*_*i*_|^*Xi*^,*Y*) = *ln*⁡Σ_*x*_*i*_|^*Xi*^,*Y*_, *V*(*y*_*j*_|^*Yj*^,*X*) = *ln*⁡Σ_*y*_*j*_;^*Yj*^,*X*_. For example, for the cardiac variable *x*_*i*_ = η, such that ^*Xi*^ = [ρπ], we have *R*(η;ρ,π,δ,θ,α,β) = *ln*⁡(Σ_η_)−*ln*⁡(Σ_η|ρπδθαβ_) [Figure 1C (right), blue + green]. In a similar way, the interaction between a target variable of a given subnetwork (brain or body) and the remaining variables in the same subnetwork is quantified as

$$R\left(x_{i};X^{i}\right)=V\left(x_{i}\right)-V\left(x_{i}|X^{i}\right),$$

(9) $$R\left(y_{j};Y^{j}\right)=V\left(y_{j}\right)-V\left(y_{j}|Y^{j}\right);$$

a graphical example with *x*_*i*_ = η is in Figure 1C (right, green). Moreover, conditional interactions can be measured to assess the link between two variables after removing the common effect that a group of other variables has on them. Here, we measure the interaction between one target variable in a subnetwork and all variables in the other subnetwork, conditioning on the remaining variables in the first subnetwork, as follows:

$$R\left(x_{i};Y|X^{i}\right)=V\left(x_{i}|X^{i}\right)-V\left(x_{i}|X^{i},Y\right),$$

(10) $$R\left(y_{j};X|Y^{j}\right)=V\left(y_{j}|Y^{j}\right)-V\left(y_{j}|Y^{j},X\right);$$

a graphical example with *x*_*i*_ = η is in Figure 1C (right, blue). We note that Eqs 9 and 10 achieve a decomposition of Eq. 8, i.e., *R*(*x*_*i*_;^*Xi*^,*Y*) = *R*(*x*_*i*_;^*Xi*^) + *R*(*x*_*i*_;*Y*|^*Xi*^) and *R*(*y*_*j*_;^*Yj*^,*X*) = *R*(*y*_*j*_;^*Yj*^) + *R*(*y*_*j*_;*X*|^*Yj*^). For instance, Figure 1C (right) depicts how the extent of common variability shared between the cardiac variable and all other physiological variables, *R*(η;π,ρ,*Y*), can be expanded as the sum of the variability the cardiac variable shares with the two other variables of the body subnetwork, *R*(η;π,ρ), and the variability that it shares with the brain subnetwork but not with the body subnetwork, *R*(η;*Y*|π,ρ).

Finally, we define a measure of the “direct” interaction between two individual physiological processes *z*_*i*_,*z*_*j*_ ∈ *Z* conditioned to all other processes in the overall network as the quantity:

(11) $$R\left(z_{i};z_{j}|Z\backslash \left\{z_{i},z_{j}\right\}\right)=V\left(z_{j}|Z\backslash \left\{z_{i},z_{j}\right\}\right)-V\left(z_{j}|Z\backslash \left\{z_{j}\right\}\right),$$

which quantifies the extent of common variability between *z_j* and *z_i* that is not shared with any other variable in the network *Z*. For instance, the direct interaction between the cardiac and respiratory variables is given by *R*(η;ρ|π,δ,θ,α,β) = *ln*⁡(Σ_η|π,δ,θ,α,β_)−*ln*⁡(Σ_η|ρ,π,δ,θ,α,β_) (Figure 1C, middle).

### Data Analysis and Statistical Analysis

All the measures presented in the previous subsection were computed from the *M* = 7 time series collected from each of the 18 subjects in the three analyzed experimental conditions (REST, MENTAL, and GAME). Moreover, for all measures involving the brain processes (vector variable *Y*), the computation was repeated, for each of the 14 scalp electrodes, extracting the *Q* = 4 brain time series δ, θ, α, and β from the EEG signal acquired on that electrode while considering the same *P* = 3 body time series (see Figure 1A). For each set of time series, the analysis was computed using the ordinary vector least squares approach to identify the linear regression models needed for the computation of the generalized partial variances in Eq. 7 and of the partial variances in Eqs 8–11.

After computation of each interaction measure, its statistical significance was tested, individually for each computation, by using a parametric Fisher statistic (Brandt and Williams, 2006) under the null hypothesis that the coefficients of the considered linear relationship are all zero (Montalto et al., 2014; Siggiridou and Kugiumtzis, 2015). In all those cases in which it is necessary to solve two different linear regression problems with scalar predicted variable, i.e., for the computation of *R*(*x*_*i*_;*Y*|^*Xi*^), *R*(*y*_*j*_;*X*|^*Yj*^) and *R*(*z*_*i*_;*z*_*j*_|*Z*\{*z*_*i*_,*z*_*j*_}), the test statistic is:

(12) $$F=\frac{\frac{R\,S\,S_{R}-R\,S\,S_{F}}{p_{F}-p_{R}}}{\frac{R\,S\,S_{F}}{N-p_{F}}},$$

where *RSS*_*R*_ and *RSS*_*F*_ are the residual sum of squares of the reduced and full regression (leading to compute the first and second *V*(⋅|⋅) terms, respectively), *p_R* and *p_F* are the number of coefficients used in the reduced and full regression, and *N* is the time series length. The interaction measure is considered statistically significant if *F* is larger than the critical value of the Fisher distribution with (*p_F-p_R*, *N-p_F*) degrees of freedom at the significance level α0.05. When it is necessary to solve only one linear regression problem, i.e., for the computation of *R*(*y*_*j*_;^*Yj*^), *R*(*y*;^*Yj*^,*X*), *R*(*x*_*i*_;^*Xi*^), and *R*(*x*_*i*_;^*Xi*^,*Y*), the *RSS*_*R*_ reduces to the variance of the predicted variable, Σ_*X*_. Lastly, for the computation of *R*(*X*;*Y*) in which *X* and *Y* are both multivariate, *RSS*_*R*_ is the generalized variance of *X*, |Σ_*X*_|, and *RSS*_*R*_ is the generalized partial variance of *X* given *Y*, |Σ|X|Y.

As regards the statistical analysis, the deviation from homogeneity of the spatial distribution of each interaction measure was assessed using the non-parametric Kruskal–Wallis test, which was also used to assess the statistical significance of the difference across conditions (REST, MENTAL, and GAME) of the median of the distribution of the measure computed over the 18 subjects, followed in this case by *post hoc* Dunn–Šidák test with correction for multiple comparisons (Šidák, 1967; Sawilowsky, 2007) to assess pairwise differences (REST vs. MENTAL, REST vs. GAME, MENTAL vs. GAME). Non-parametric tests were used because the hypothesis of normality of the distribution of each measure was rejected according to the Anderson–Darling test (Anderson and Darling, 1952).

## Results

**FIGURE 2:**
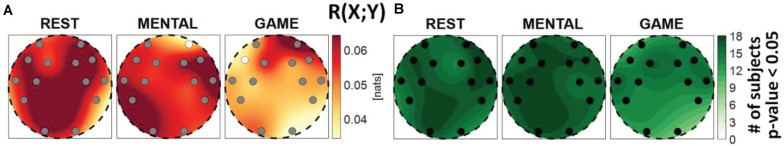
Spatial distribution of **(A)** the median multivariate interaction between brain and body, *R*(X; Y), and **(B)** the number of subjects with statistically significant values of the index, in the three analyzed conditions (REST, MENTAL, and GAME). Markers are located at EEG electrode positions and in **(A)** are colored according to the results of statistical analysis (white: *p* < 0.05 MENTAL vs. REST or GAME vs. REST).

**FIGURE 3:**
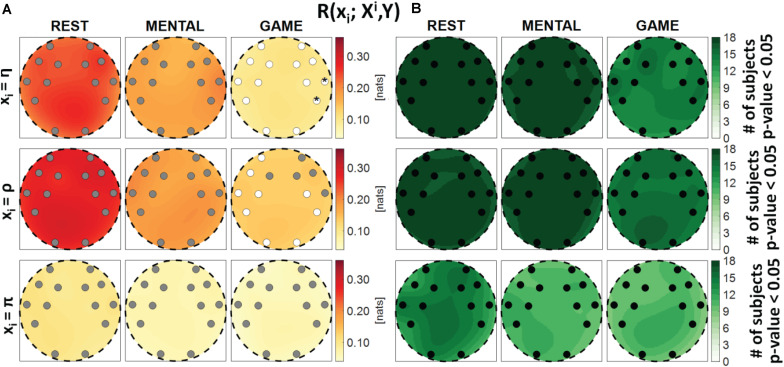
Spatial distribution over the scalp of **(A)** the median multivariate interaction between a target *x_i* of the body subnetwork and all remaining variables, *R*(*x*_*i*_;^*Xi*^,*Y*), and **(B)** the number of subjects with statistically significant values of the index, in the three analyzed conditions (REST, MENTAL, and GAME); the index is computed with target corresponding to the cardiac process η (upper row panels), to the respiratory process ρ (middle row panels), and to the cardiovascular process π (lower row panels). Markers are located at EEG electrode positions and in **(A)** are colored according to the results of statistical analysis (white: *p* < 0.05 MENTAL vs. REST or GAME vs. REST). Asterisk (*) on an electrode indicates *p* < 0.05 MENTAL vs. GAME.

**FIGURE 4:**
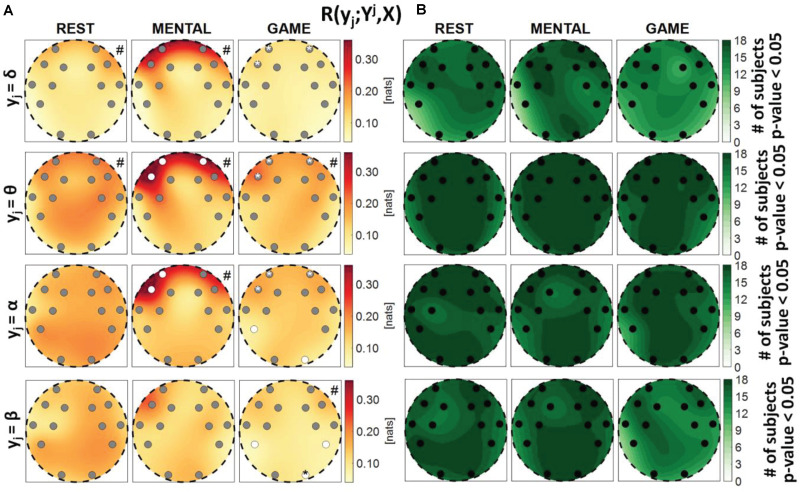
Spatial distribution over the scalp of **(A)** the median multivariate interaction between a target *y_j* of the brain subnetwork and all remaining variables, *R*(*y*_*j*_;^*Yj*^,*X*), and **(B)** the number of subjects with statistically significant values of the index, in the three analyzed conditions (REST, MENTAL, and GAME); the index is computed with target corresponding to the EEG power band processes δ,θ,α,β (from upper to lower row panels). Markers are located at EEG electrode positions and in **(A)** are colored according to the results of statistical analysis (white: *p* < 0.05 MENTAL vs. REST or GAME vs. REST). Asterisk (*) on an electrode indicates *p* < 0.05 MENTAL vs. GAME. Hash symbols indicate results of Kruskal–Wallis test (#: *p* < 0.05, non-homogeneity of the spatial distribution).

**FIGURE 5:**
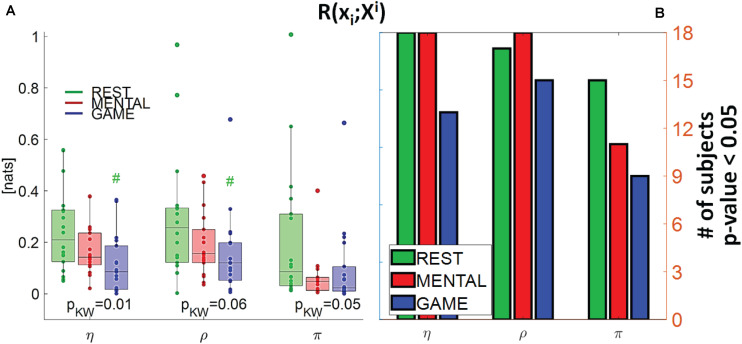
**(A)** Distributions of the multivariate interaction between a target *x_i* of the body subnetwork and the two other variables of the same subnetwork, *R*(*x*_*i*_;^*Xi*^), and **(B)** number of subjects with statistically significant values of the index, in the three analyzed conditions (REST, MENTAL, and GAME); the index is computed with target corresponding to the cardiac, respiratory, and cardiovascular processes η,ρ, and π. In **(A)**, p_*KW*_ indicates results of Kruskal–Wallis test, while hash symbols indicate a *p*-value lower than 0.05 obtained using post-hoc test for the analysis between REST and the considered condition (#: *p* < 0.05 MENTAL vs. REST or GAME vs. REST).

**FIGURE 6:**
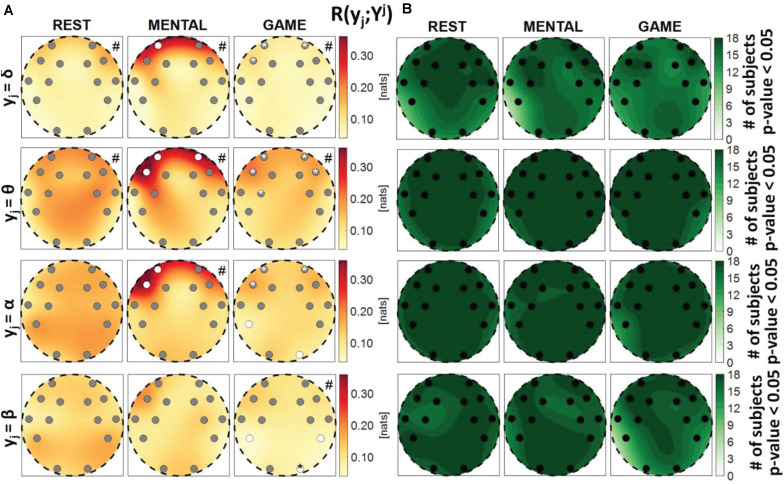
Spatial distribution over the scalp of **(A)** the median multivariate interaction between a target *y_j* of the brain subnetwork and the three other variables in the same subnetwork, *R*(*y*_*j*_;^*Yj*^), and **(B)** number of subjects with statistically significant values of the index, in the three analyzed conditions (REST, MENTAL, and GAME); the index is computed with target corresponding to the EEG power band processes δ,θ,α,β (from upper to lower row panels). Markers are located at EEG electrode positions and in **(A)** are colored according to the results of statistical analysis (white: *p* < 0.05 MENTAL vs. REST or GAME vs. REST). Asterisk (*) on an electrode indicates *p* < 0.05 MENTAL vs. GAME. Hash symbols indicate results of Kruskal–Wallis test (#: *p* < 0.05, non-homogeneity of the spatial distribution).

**FIGURE 7:**
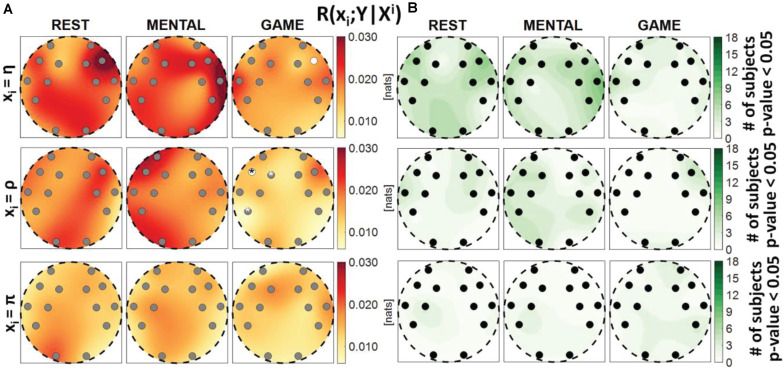
Spatial distribution over the scalp of **(A)** the median multivariate interaction between a target *x_i* of the body subnetwork and all variables of the brain subnetwork, conditioned on the two remaining variables of the body subnetwork, *R*(*x*_*i*_;*Y*|^*Xi*^), and **(B)** the number of subjects with statistically significant values of the index, in the three analyzed conditions (REST, MENTAL, and GAME); the index is computed with target corresponding to the cardiac process η (upper row panels), to the respiratory process ρ (middle row panels), and to the cardiovascular process π (lower row panels). Markers are located at EEG electrode positions and in **(A)** are colored according to the results of statistical analysis (white: *p* < 0.05 MENTAL vs. REST or GAME vs. REST). Asterisk (*) on an electrode indicates *p* < 0.05 MENTAL vs. GAME. Hash symbols indicate results of Kruskal–Wallis test (#: *p* < 0.05, non-homogeneity of the spatial distribution).

**FIGURE 8:**
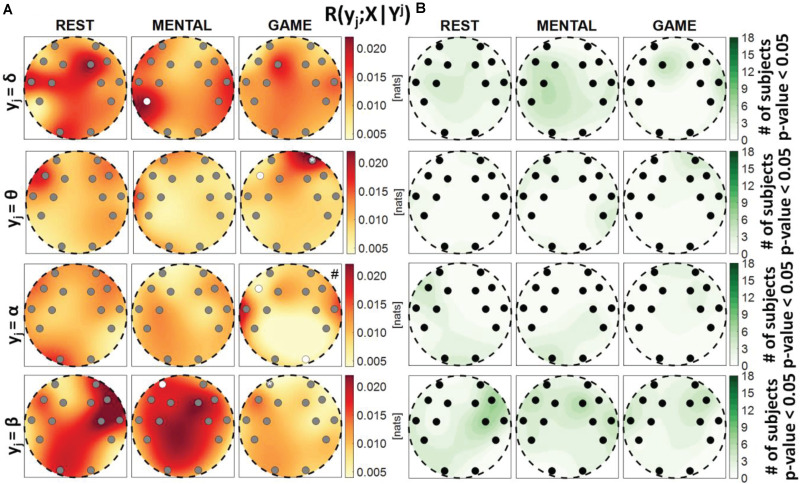
Spatial distribution over the scalp of **(A)** the median multivariate interaction between a target *y_j* of the brain subnetwork and all variables of the body subnetwork, conditioned on the three remaining variables of the brain subnetwork, *R*(*y*_*j*_;*X*|^*Yj*^), and **(B)** the number of subjects with statistically significant values of the index, in the three analyzed conditions (REST, MENTAL, and GAME); the index is computed with target corresponding to the EEG power band processes δ,θ,α,β (from upper to lower row panels). Markers are located at EEG electrode positions and in **(A)** are colored according to the results of statistical analysis (white: *p* < 0.05 MENTAL vs. REST or GAME vs. REST). Asterisk (*) on an electrode indicates *p* < 0.05 MENTAL vs. GAME. Hash symbols indicate results of Kruskal–Wallis test (#: *p* < 0.05, non-homogeneity of the spatial distribution).

Results are presented showing the median values, across the subjects, of the various interaction measures in the three considered conditions (REST, MENTAL, and GAME). The spatial distribution of each measure is obtained performing the analysis at every EEG electrode location, and is represented with color-coded values carrying out an interpolation over the schematic of the scalp. In addition, figures show the results of the statistical significance analysis, reporting the number of subjects for which the measure was found to be significantly larger than zero according to the Fisher F-test. We refer the reader to the Supplementary Material for the complete table of results in terms of median MI values, *p*-values of Kruskal–Wallis and *post hoc* pairwise comparison test, and of number of subjects with statistically significant MI according to Fisher F-test for the Figures 2–8.

### MI Between Each Subnetwork as a Whole

Figure 2 shows the median MI index *R*(*X*;*Y*) computed between the brain and body subnetworks (Figure 2A), and the number of subjects which showed statistically significant MI (Figure 2B), mapped across the scalp in the three analyzed conditions. The index *R*(*X*;*Y*)can be thought as a measure of the overall connectivity between the body and brain subnetworks, each one considered as a whole. In almost all subjects and especially during the REST and MENTAL conditions, the two subnetworks share statistically significant amounts of information at all the EEG electrodes positions (Figure 2B). In each condition, the Kruskal–Wallis test showed homogeneity (*p*-value > 0.05) for the spatial distribution of the MI index (the visually heterogeneous patterns in Figure 2A may be due to interpolation effects due to the limited number of non-uniformly distributed electrodes). The overall connectivity tends to decrease going from REST to MENTAL and then to GAME (Figure 2A); compared to REST, the decrease is statistically significant for the AF4 frontal electrode during MENTAL and for the F7 electrode during GAME.

### MI Between a Target and All Other Processes in the Brain–Body Network

Figure 3 reports the spatial distribution on the scalp of the median values of the MI between a target *i* of the body subnetwork and all other processes, i.e., *R*(*x*_*i*_;^*Xi*^,*Y*) (a), alongside with the number of subjects which showed statistically significant MI according to the F-test (b). This measure evaluates the degree of connectivity between the considered body process and all other processes in the overall network. Considering the cardiac variable η or the respiratory variable ρ as the target, the MI value was found to be high and statistically significant in all subjects during REST and MENTAL (with a slight decrease in the median values during MENTAL), while it decreased markedly in magnitude during GAME, also resulting statistically significant in a lower number of subjects. The decrease from REST to GAME was statistically significant at all locations with target η, and at the locations of the electrodes AF3, F7, T7, FC5, FC6, P7, P8, O1, and O2 with target ρ. On the contrary, when the cardiovascular process π was taken as the target, the MI value was low and was significant in a smaller number of subjects (around 50%), without displaying any significant variations across conditions. The Kruskal–Wallis test showed homogeneity (*p*-value > 0.05) for spatial distributions of*R*(*x*_*i*_;^*Xi*^,*Y*) in all the cases. These results denote a high degree of connectivity between the cardiac and respiratory processes and the other network processes, decreasing with the GAME task, and an overall low connectivity for the cardiovascular process.

Figure 4 reports the spatial distribution on the scalp of the median values of the MI between a target *j* of the brain subnetwork and all other processes, i.e., *R*(*y*_*i*_;^*Yj*^,*X*) (a), alongside with the number of subjects which showed statistically significant MI according to the F-test (b). The measure evaluates the connectivity between the considered brain rhythm and all other processes in the overall network. The MI relevant to the δ, θ, and α brain variables showed a tendency to increase, when assessed for electrodes located in the frontal area of the scalp, during the mental arithmetic condition compared to the resting state, and to return to baseline values during the serious game condition. The index *R*(*y*_*i*_;^*Yj*^,*X*) increased significantly at the AF3, AF4, and F7 electrodes for θ and at the AF3 and F7 electrodes for α, moving from REST to MENTAL, reflecting an increased interaction between such rhythms and the whole network during mental workload in the frontal region, and decreased significantly at AF3, AF4, and F7 electrodes for δ, θ, and α moving from MENTAL to GAME; the decrease was statistically significant also at the left parietal P7 and right occipital O2 electrodes when *y*_*j*_ = α and comparing GAME to REST. A different behavior was observed taking the process β as target, with no variations of the median MI values going from REST to MENTAL, a decrease at the P7, P8, and O2 electrodes going from REST to GAME, and a decrease at the O2 electrode going from GAME to MENTAL; this suggests a decreased connectivity between the β rhythm and all others localized to the parietal and right occipital regions. The Kruskal–Wallis test showed a heterogeneous spatial distribution of *R*(*y*_*i*_;^*Yj*^,*X*) (*p*-value < 0.05) when *y*_*i*_ = θ during all three conditions, when *y*_*i*_ = δ during REST and MENTAL, when *y*_*i*_ = α during MENTAL, and when *y*_*i*_ = β during GAME. The F-test showed statistically significant values of *R*(*y*_*i*_;^*Yj*^,*X*) for almost all subjects when *y*_*i*_ = θ, *y*_*i*_ = α, and *y*_*i*_ = β (in particular during REST and MENTAL), while it was significant for a lower number of subjects (around 60%) when *y*_*i*_ = δ (especially during GAME).

### MI Between a Target and All Other Processes in the Brain or Body Subnetwork

**FIGURE 9:**
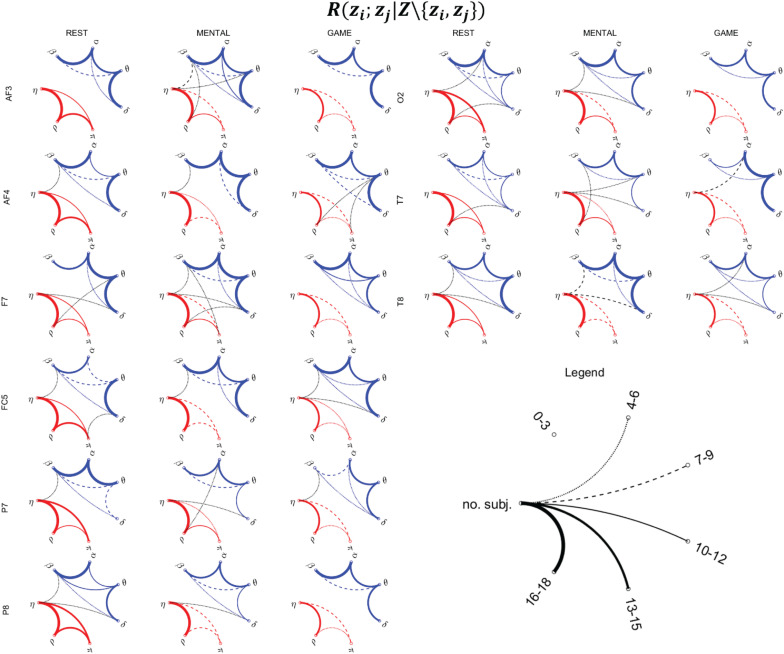
Topological representation of the interaction between pairs of nodes *z_i* and *z_j* of the physiological network (*z*_*i*_,*z*_*j*_ ∈ *Z* = {η,ρ,π,δ,θ,α,β}), provided by the statistically significant values of the conditional MI measure *R*(*z*_*i*_;*z*_*j*_|*Z*\{*z*_*i*_,*z*_*j*_}), during the three considered states (REST, MENTAL, and GAME). Thickness of the lines is proportional to the number of subjects for which the corresponding link is statistically significant (*p* < 0.05, Fisher’s F-test). Red, blue, and black lines denote the links relevant to body–body, brain–brain, and brain–body interactions.

Figure 5 depicts the distribution of the MI between a target in the body subnetwork and the two other variables belonging to the same subnetwork, *R*(*x*_*i*_;^*Xi*^), in the three conditions, together with the number of subjects with statistically significant MI. This index assesses the internal connectivity of the body subnetwork, measured between one process and the two others, while pairwise “direct” connectivity can be inferred from Figure 9. For each target node, its interaction within the body subnetwork was found high and significant at REST and decreased progressively during the MENTAL and GAME conditions. The decrease of MI values from REST to GAME is statistically significant for η and ρ taken as targets. The values of *R*(*x*_*i*_;^*Xi*^) computed with *x*_*i*_ = η and *x*_*i*_ = ρ were statistically significant in almost all subjects during REST and MENTAL, and decreased slightly during GAME; when *x*_*i*_ = π, the statistical significance was lower in all conditions and reached the minimum of 50% of subjects during GAME. Overall, these results suggest a strong connectivity within the body subnetwork, mainly arising from cardiorespiratory interactions and declining during mental workload.

Figure 6 depicts the distribution of the MI between a target in the brain subnetwork and the three other variables belonging to the same subnetwork, *R*(*y*_*j*_;^*Yj*^), as well as the number of subjects with statistically significant MI. This index assesses the connection of the considered brain rhythm with all the others taken together, while the pairwise connectivity between rhythms can be inferred from Figure 9. For this measure, results are similar to those obtained for the global measure *R*(*y*_*j*_;^*Yj*^,*X*), showing a tendency of the measure to increase from REST to MENTAL in the frontal region of the scalp (statistically significant at the AF3, AF4, and F7 electrodes when *y*_*j*_ = θ, at the AF3 and F7 electrodes when *y*_*j*_ = α, and at the AF3 electrode when *y*_*j*_ = δ), and a tendency to decrease in the same region moving from MENTAL to GAME (significant for AF3, AF4, and F7 when *y*_*j*_ = δ and *y*_*j*_ = α, and also for F8 when *y*_*j*_ = θ); other significant changes for *y*_*j*_ = α involved the P7 and O2 electrodes when comparing GAME and REST, and for *y*_*j*_ = θ the electrode FC5 when comparing GAME and MENTAL. These results indicate an increased connectivity of δ, α, and especially θ rhythms with all the others during mental workload in the frontal region, and a decreased connectivity of α with all the others during GAME in the left parietal and right occipital zones. Different trends were shown when *y*_*j*_ = β: the MI was substantially unchanged from REST to MENTAL, and decreased during GAME (with significant changes at the electrodes P7, P8, and O2 when compared to REST, and at O2 when compared to MENTAL), thus showing a decreased connectivity of β rhythm with all the others during GAME in the parietal and right occipital regions. The Kruskal–Wallis test showed spatial inhomogeneity (*p*-value < 0.05) with regard to δ and θ power in all the three conditions, only during MENTAL with regard to α power, and only during GAME with regard to β power. According to the F-test, the interaction values were statistically significant in the large majority of subjects for all measures and conditions.

### Conditional MI Between a Target in a Subnetwork and the Whole Other Subnetwork

Figure 7 reports the spatial distribution on the scalp of the median values of the conditional MI between a target *i* of the body subnetwork and the whole brain subnetwork, given the remaining variables in the body subnetwork, i.e., *R*(*x*_*i*_;*Y*|^*Xi*^) (Figure 7A), alongside with the number of subjects which showed statistically significant conditional MI according to the F-test (Figure 7B). This measure evaluates the strength of the connection of a body process with all the brain rhythms, after conditioning on effects of the other body processes. Contrary to the MI measures previously analyzed, the conditional MI showed overall lower values, as *R*(*x*_*i*_;*Y*|^*Xi*^) was on average an order of magnitude smaller than *R*(*x*_*i*_;^*Xi*^), and weaker statistical significance, as the F-test rejected the null hypothesis only for few subjects (always less than 50%) in all the conditions and electrodes. The conditional MI showed a tendency to decrease during GAME when *x*_*i*_ = η (significantly lower values at F8 compared to REST) and when *x*_*i*_ = ρ (significantly lower values at F7 compared to REST, and at F7, F3, and P7 compared to MENTAL), while it was uniformly low when *x*_*i*_ = π. The Kruskal–Wallis test showed homogeneity (*p*-value > 0.05) for the spatial distributions of*R*(*x*_*i*_;*Y*|^*Xi*^) in the three considered conditions.

Figure 8 reports the spatial distribution on the scalp of the median values of the conditional MI between a target *j* of the brain subnetwork and the whole body subnetwork, given the remaining variables in the brain subnetwork, i.e., *R*(*y*_*j*_;*X*|^*Yj*^) (Figure 8A), alongside with the number of subjects which showed statistically significant conditional MI according to the F-test (Figure 8B). This measure evaluates the strength of the connection of a brain rhythm with all the body processes, after conditioning on effects of the other brain rhythms. Also in this case, the values of *R*(*y*_*j*_;*X*|^*Yj*^) were much weaker than those of the unconditional measure *R*(*y*_*j*_;^*Yj*^) and exhibited markedly lower statistical significance (compare Figure 8 with Figure 6). The conditional MI showed a tendency to increase during MENTAL when *y*_*j*_ = δ and when *y*_*j*_ = β (significantly higher values compared to REST, respectively, at P7 and at AF3), and to decrease during GAME when *y*_*j*_ = θ and when *y*_*j*_ = α (significantly lower values compared to REST, respectively, at F7 and at F7, O2); an increase from MENTAL to GAME was observed at AF4 when *y*_*j*_ = θ. These results, together with those of Figure 7, highlight the presence of weak connectivity between the brain and body processes, with no precise trends in terms of spatial localization and just a few statistically significant variations during MENTAL or GAME. The Kruskal–Wallis test showed homogeneity (*p*-value > 0.05) for the spatial distributions of*R*(*y*_*j*_;*X*|^*Yj*^) in almost all of the cases, except than the case *y*_*j*_ = α during GAME.

### Direct Interactions Between Pairs of Processes Based on Conditional MI

Figure 9 reports the network representation of the direct interactions between pairs of variables of the physiological network across the three analyzed conditions, depicted on the basis of the conditional MI measure *R*(*z*_*i*_;*z*_*j*_|*Z*\{*z*_*i*_,*z*_*j*_}). This measure evaluates the pairwise connectivity between two processes in the context of all other processes in the whole physiological network. In the figure, networks are constructed counting the subjects for which the measure was statistically significant, and for visualization purposes are reported for the subset of the scalp electrodes for which most significant variations were observed in the previous analyses (frontal: AF3, AF4, F7; central: FC5; parietal: P7, P8; occipital: O2; temporal: T7, T8).

The network analysis allows to investigate the topological structure underlying the MIs detected previously, as well as their changes across conditions. As regards the body subnetwork (red links), the topology is quite consistent across electrodes for any considered experimental condition. At REST, strong interconnections are observed between the η and ρ nodes, and significant (though generally weaker) connections are also observed between η and π and between π and ρ. During MENTAL, the connection η–ρ remains significant in almost all subjects, while the two other links (η–π and π–ρ) are generally less evident. The weakening of the links in the body subnetwork is even more evident during GAME, involving also a decrease in the number of the connections between η and ρ.

Analyzing the brain subnetwork (blue links), we found that it is fully connected (i.e., it shows absence of isolated nodes) for any scalp electrode and experimental condition. The most evident connections are those involving the pairs of nodes δ–θ, θ–α, and α–β, while the connections δ–α, δ–β, and θ–β are weaker and less consistent across spatial locations. This topology is present in all conditions at REST and persists in the other conditions, even though with some noticeable anatomical variations moving from one condition to another. During MENTAL, the connections δ–θ, θ–α, and α–β were reinforced in the frontal areas of the scalp (AF3, AF4, F7, FC5) and in the right temporal area (T8). A slightly larger variability in the topology was observed during GAME, with connection strength similar to REST although with some local difference (e.g., emergence of θ–β connections at F7, decrease of α–β connections at P7, increase of δ–θ connections at T7, and decrease at T8).

Brain–body interactions (black links) are less evident and more sparse, supporting in terms of the fully multivariate measure *R*(*z*_*i*_;*z*_*j*_|*Z*\{*z*_*i*_,*z*_*j*_}) the results of Figures 7, 8 where a limited number of significant values of *R*(*x*_*i*_;*Y*|^*Xi*^) and *R*(*y*_*j*_;*X*|^*Yj*^) were observed. Though weak, interactions between the brain and body subnetwork were almost always detected (the two subnetwork were isolated only at AF3 during REST, and at AF3, F7, P8, O2 during GAME). Such interactions were mostly involving the η node of the body subnetwork (in 29 out of the 40 brain–body connections shown in Figure 9), often linked to the β node of the brain subnetwork (in 13 cases), or the δ node of the brain subnetwork (14 connections), and only sporadically the remaining nodes. Overall, brain–body connections increased moving from REST to MENTAL (from 13 to 19 links shown in Figure 9) and decreased during GAME (eight links); the scalp electrodes where this behavior was more striking are located in the frontal (AF3, F7) and temporal (T7, T8) areas.

## Discussion

The main results of this work can be summarized as follows: (a) the brain and body subnetworks of the human physiological network exhibit significant degrees of internal and reciprocal interaction; (b) internal interactions (brain–brain and body–body) are predominant, confirming the existence of significantly correlated variations in the amplitude of the different brain waves on one side (Lin et al., 2020), and of cardiovascular and cardiorespiratory interactions on the other side (Porta et al., 2012; Schulz et al., 2013); (c) cardiorespiratory interactions are the predominant form of interaction within the analyzed body subnetwork, and decrease significantly during sustained attention (and less evidently during mental stress); (d) brain–brain interactions are sustained by a quite consistent topological structure, and are significantly stronger in the frontal scalp areas during mental stress; (e) brain–body interactions are weaker than within-subnetwork interactions, but are often statistically significant and are modulated by the physiological state, being stronger during the mental stress task and weaker during the sustained attention task.

Our results suggest the presence of strong interactions within and between the brain and body subnetworks which vary according to the stress level elicited by the adopted protocol, as highlighted by the analysis of the MI measure *R*(*X*;*Y*) (Figure 2). This finding is in line with those of several investigations in the field of network physiology showing that significant degrees of interaction within and between organ systems sustain the physiological regulation in different physiological states, e.g., including sleep stages (Ako et al., 2003; Bashan et al., 2012; Bartsch et al., 2015; Lin et al., 2020) or physiological stress (Faes et al., 2017b; Valente et al., 2018; Krohova et al., 2019). Nevertheless, exploiting the decomposition of the overall MI measure into measures eliciting the correlations relevant to a single target variable and selected groups of other variables, it has been possible to infer that the interactions within each subnetwork prevail over brain–body interactions. This fact is documented by the low absolute values and fraction of subjects with statistically significant interaction observed for the conditional MI measures *R*(*x*_*i*_;*Y*|^*Xi*^) and *R*(*y*_*j*_;*X*|^*Yj*^) (see Figures 7, 8), as well as from the similar trends obtained for MI (Figures 3, 5) and conditional MI measures (Figures 4, 6). Weaker interactions between the brain and body subnetworks were observed in the same experimental settings also in recent studies performing dynamic analyses (Zanetti et al., 2019a; Antonacci et al., 2020).

The interactions occurring within the body subnetwork formed by cardiac, cardiovascular, and respiratory dynamics (Figure 5 and red links in Figure 9) were remarkable and quite consistent across conditions, evidencing a predominance of cardiorespiratory coupling and a weakening during mental stress and particularly during sustained attention. The strong link between the cardiac and respiratory variables, corresponding to the heart period and respiratory amplitude time series, is due to the respiratory sinus arrhythmia (RSA), a well-known physiological mechanism whereby the breathing activity modulates the variability of the heart rate (Yasuma and Hayano, 2004; Ben-Tal et al., 2012; Porta et al., 2012; Krohova et al., 2019). Our results are in agreement with those obtained in previous works using different and more sophisticated techniques, e.g., in Zanetti et al. (2019a) computing the information the information exchanged dynamically between heart period and respiration, and in Krohova et al. (2019) applying multiscale entropy methods. In the latter study, the weakening of the influence of respiration on heart rate has been ascribed to the inhibition of parasympathetic activity provoked by stress challenges and, when compared to other stressors like postural changes, to the lack of activation of baroreflex-mediated RSA mechanisms. We also found that the cardiovascular variable analyzed here, i.e., the PAT, strongly interacts with the cardiac period and the respiration amplitude. This link is mostly probably due to the known influence of heart rate on stroke volume and blood pressure that in turns varies the PAT, which is also influenced by respiration (Drinnan et al., 2001; Wang et al., 2014); the mechanism is such that respiration affects the intra-thoracic pressure provoking changes in blood pressure and then also heart rate, with PAT variations following some beats later (Cavalcanti, 2000; Drinnan et al., 2001).

Considering the interactions of the processes belonging to the brain subnetwork (Figure 6), our results highlight a marked increase in the frontal region occurring during the mental arithmetic task (but not during the attention task) for the links involving the δ, θ, and α EEG power time series. This finding supports from the point of view of connectivity between different brain rhythms the well-known fact that mental arithmetic tasks and operations with numbers produce an activation of specific prefrontal cortical areas (Inouye et al., 1993; Menon, 2010; Arsalidou and Taylor, 2011; Friedrich and Friederici, 2013). Moreover, the decrease observed in the parietal and right occipital regions moving from rest to serious game when the MI term *R*(*y*_*j*_;^*Yj*^) was computed for the α and β EEG rhythms is in accordance with previous findings in the literature showing that parietal cortical regions, mainly in the right hemisphere, are involved in sustained attention tasks (Lawrence et al., 2003; Molteni et al., 2007; Klimesch, 2012; Saalmann et al., 2012; Behzadnia et al., 2017; Mitko et al., 2019); the modulation of EEG rhythms during sustained attention was previously observed regarding high-frequency waves (in the β and gamma ranges) in Molteni et al. (2007), with changes localized mostly in the right hemisphere and in the parietal region, and regarding the α rhythm in Behzadnia et al. (2017), showing that a greater decrease in the α power is associated with better performance during the task. Our study goes beyond the above described findings, also showing that the observed changes in the coupling strength between brain wave dynamics are supported by the topology of the brain–brain network and to its reorganization during mental stress and sustained attention. Remarkably, structured reorganizations of the connectivity and topology of physiological networks consequent to transitions across different physiological states have been previously reported in the context of sleep analysis (Bashan et al., 2012; Faes et al., 2015b; Lin et al., 2020). Bashan et al. (2012) demonstrated that the strength of brain–brain links is high during light sleep and deep sleep, and is lower during rapid eye movement (REM) sleep; Lin et al. (2020) reported strong β–α and θ–α links in awake subjects and when significant positive correlation is present between a pair of brain waves; in Faes et al. (2015b), strong dynamical interactions along the directions β->α and δ->θ were revealed during sleep employing time-lagged causality measures such as Granger causality and transfer entropy.

The analysis of brain–heart interactions, evidenced particularly by the network topology in Figure 9, documented an increased connectivity between the brain and body subnetworks during mental stress (especially in the frontal scalp areas), and a reduction during sustained attention when conditions of isolation of the two sub-networks were often encountered (e.g., at electrodes AF3, F7, P8, and O2). The increased brain–body connectivity during the mental arithmetic task is likely related to the widely studied compensatory responses co-occurring in the central and ANSs to the internal and environmental stimuli evoked by stress (see, e.g., Silvani et al., 2016 for a review on the topic). As regards the use of multivariate time series analysis techniques, findings similar to those reported here were obtained performing a dynamic analysis based on Granger causality in Zanetti et al. (2019a); moreover, stronger bidirectional interactions between brain and heart dynamics were reported during emotional elicitation (Greco et al., 2019). As regards the nature of brain–body interactions, we find that those occurring more frequently are involving the variability of the heart period and of the β EEG waves. This finding is in accordance with what reported in Mather and Thayer (2018) where it is stated that oscillations in heart rate modulate brain oscillatory activities, especially in brain regions associated with emotion regulation, which can lead to enhanced functional connectivity. Other studies, mostly related to sleep analysis, also suggest the existence of relations between EEG rhythms and HRV arising from common effects driven by the ANS (Ako et al., 2003; Faes et al., 2014; Kuo et al., 2016; Dzhebrailova et al., 2017). In particular, the β waves seem to play a main role in mediating brain–heart interactions, likely due to their dependence on autonomic arousals and sympathetic activation (Faes et al., 2014; Kuo et al., 2016). In addition, considering that cardiorespiratory interactions are typically very strong, an indirect effect (i.e., an effect mediated by RSA) of respiration on the brain subnetwork seems also plausible. Such an effect is also supported by evidences about the rhythmic modulation of the neuronal activity of the neocortex exerted by respiration-locked sensory inputs (Heck et al., 2017; Varga and Heck, 2017). On the contrary, the interaction between π and the other variables is quite limited (as demonstrated by the low MI values in Figure 3A and the few connections in Figure 9). This is also in agreement with recent results (Pernice et al., 2019d; Zanetti et al., 2019a) obtained using information-theoretic measures, suggesting a limited coupling between pulse wave velocity in the cardiovascular system and brain dynamics.

Methodologically, the results of this work highlight the usefulness of the proposed MI measure to investigate the functional connection between different subnetworks in the human body. The MI measure under certain assumptions is also directly proportional to mutual information (see Appendix), and this is useful to allow comparisons with other previous works in the field, since information-theoretic-based measures have already been used in the past for this aim (Barnum et al., 2010; Faes et al., 2014, 2017a,b; Barrett, 2015). For example, in our previous work (Zanetti et al., 2019a) we have investigated the information generated, stored, and transferred among different nodes in a physiological network taking into account only one electrode, while in Pernice et al. (2019b), we have carried out a multilevel stress assessment based on the concept of network physiology using time-domain measures (mean and standard deviation) and self-entropy. Also, in Antonacci et al. (2020), we have applied a more sophisticated technique consisting of a penalized regression performed through the Least Absolute Shrinkage and Selection Operator (LASSO) before calculating measures of information dynamics. All these approaches are dynamic, meaning that they account for time-lagged interactions. Compared to such approaches, the MI measures proposed here can be defined “static,” since only instantaneous (zero-lag) interactions are taken into account. Static analysis in some sense subsumes dynamic analysis, since time lagged effect typically determine zero-lag ones; moreover, the performed zero-lag correlation analysis is easier to implement and computationally efficient. While instantaneous or-single lag interactions are the basis of the main studies in the field of network physiology (Bashan et al., 2012; Lin et al., 2020), in this study, we have extended their investigation to the multivariate case, allowing the study of interactions between groups of sub-systems (through the MI measures involving blocks of variables) and the distinction between direct and indirect/mediated connections (through conditional MI measures). Our results document how this approach leads to describe exhaustively not only the interactions occurring between different subnetworks (brain–body), but also those occurring internally in a subnetwork (brain or body).

The main limitation of the current study consists in the fact that the analysis of EEG signals has been carried out on a scalp-level, as previously stated in Section “Time Series Extraction.” We are aware that recent studies have highlighted that particular care should be assumed making inferences about brain regions since EEG scalp level connectivity does not permit a perfectly reliable interpretation of interacting brain areas as they can be corrupted by volume conduction effects or by confounding factors (Lai et al., 2018; Reid et al., 2019; Van de Steen et al., 2019). However, neural time series obtained starting from the oscillations recorded on the scalp—even if affected by confounding factors—can still represent a starting point for estimating brain network interactions (Reid et al., 2019). From this point of view, the analysis carried out in this work represents a first step to be confirmed in the future using source-reconstructed signals (Van de Steen et al., 2019), or even exploiting frameworks for the computation of source connectivity measures directly from scalp recordings (Kotiuchyi et al., 2020). Other limitations of the current study consist in the relatively small number of subjects analyzed, in the possibility of a not so-clear distinction between the elicited level stress evoked by GAME and MENTAL situations which may affect the obtained results and in the fact that blood pressure was not acquired on the subjects, which could give additional useful physiological indications.

## Conclusion

The aim of this work was to extend the analysis of functional brain–body interactions based on simple correlation tools to the multivariate case, allowing to dissect such interactions into contributions originated within and between the two physiological districts. Taken together, the proposed measures of “MI” elicit transitions across different physiological states as well as spatial features, and constitute a tool easy to implement and with low computational cost. Practical and clinical applications of this tool range from a better understanding of the links and working principles of central and autonomic neural regulation (Silvani et al., 2016), or of the physiological mechanisms underlying stressful conditions (Dimsdale, 2008), to the real-time and automatic classification in real-life scenarios using non-invasive or wearable devices (Jovanov, 2019; Pernice et al., 2019c; Vinciguerra et al., 2019).

Future developments consist in the implementation of a more complete protocol able to elicit other different levels of mental stress to better investigate on the changes in the strength of the interactions between brain and peripheral subnetworks. Such protocol should also include intermediate resting phases between stressful situations to assess whether elicited stress still produces effects during time in a consequent resting phase. Future methodological work is also envisaged regarding: (a) a thorough validation on simulations of the MI measures presented here performed also through a direct comparison with more sophisticated analysis techniques including the use of time-delayed techniques employing tools of information dynamics to retrieve directional information (Faes et al., 2014) and of non-linear model free entropy estimators (Faes et al., 2015a); (b) the frequency-specific decomposition of the proposed measures (e.g., following Faes et al., 2020) to investigate how MIs can reflect oscillatory rhythms with specific physiological meaning; and (c) the analysis on source-reconstructed signals to obtain better anatomically-localized estimates of the strength and topology of brain–body interactions (Lai et al., 2018; Van de Steen et al., 2019; Kotiuchyi et al., 2020).

## Data Availability Statement

The data are publicly available at the following link: http://www.lucafaes.net/its.html (“stress data for the scripts Example_BrainBodyStress”).

## Ethics Statement

The studies involving human participants were reviewed and approved by “Comitato Etico per la Sperimentazione con l’essere vivente dell’Università degli Studi di Trento”, via Calepina 14, 38122 Trento, Italy. The patients/participants provided their written informed consent to participate in this study.

## Author Contributions

LF contributed to conceptualization and supervision. LF, RP, and DM contributed to methodology. RP and LF contributed to software and validation. MZ and GN contributed to data curation. RP and YA contributed to writing—original draft preparation. LF and AB contributed to writing—review and editing. RP, DM, and YA contributed to visualization. GN contributed to funding acquisition. All authors have read and agreed to the published version of the manuscript.

## Conflict of Interest

The authors declare that the research was conducted in the absence of any commercial or financial relationships that could be construed as a potential conflict of interest.

## Appendix

In this Appendix, we report the derivation of the alternative definitions of squared correlation for two scalar variables (Eqs 2 and 3) and their generalization in the case of vector variables (Eq. 5), and we draw the connection between the interaction measures and information measures.

In the case of scalar random variables *x* and *y*, the covariance of *W* = [*x**y*] is the 2 × 2 matrix

(A.1) $$\Sigma_{W}=\left[\begin{array}{cc} \Sigma_{x} & \Sigma_{x;y}\\ \Sigma_{y;x} & \Sigma_{y} \end{array}\right],$$

and its determinant is |Σ_[*x**y*]_|=|Σ_*W*_|$=\Sigma_{x}\,\Sigma_{y}-\Sigma_{x;y}^{2}$. Then, Eq. 2 follows easily inserting $\Sigma_{x;y}^{2}=\Sigma_{x}\,\Sigma_{y}-\left|\Sigma_{W}\right|$ in Eq. 1. Moreover, relating *x* and *y* through the linear regression model *x* = *a**y* + *b* + *u*, under the typical assumptions that *u* has zero mean and is uncorrelated with *y*, the variance of *x* and the covariance between *x* and *y* can be written, respectively, as Σ_*x*_ = *𝔼*[^(*x*−*m*_*x*_)2^]=*a*^2^Σ_*y*_ + Σ_*u*_, and Σ_*x*;*y*_ = *𝔼*[(*x*−*m*_*x*_)(*y*−*m*_*y*_)]=*a*Σ_*y*_, which combined together yield $\Sigma_{x;y}^{2}=\Sigma_{x}\,\Sigma_{y}-\Sigma_{u}$. This latter expression inserted into Eq. 1 yields Eq. 3.

Extending the above derivation to the multivariate case, the vector variables *X* and *Y* are related through the multivariate linear regression *X* = *YA* + *B* + *U* from which, assuming *m*_*U*_ = 0 and *𝔼*[^*Y**T*^*U*]=0, the covariance of *X* and the cross-covariance between *Y* and *X* can be written, respectively, as Σ_*X*_ = *𝔼*[^(*X*−*m*_*X*_)*T*^(*X*−*m*_*X*_)]=^*A**T*^Σ_*Y*_*A* + Σ_*U*_ and Σ_*Y*;*X*_ = *𝔼*[^(*Y*−*m*_*Y*_)*T*^(*X*−*m*_*X*_)]=Σ_*Y*_*A*, which combined together yield $\Sigma_{U}\equiv \Sigma_{X}-\Sigma_{X;Y}\,\Sigma_{Y}^{-1}\,\Sigma_{Y;X}$. Moreover, considering that the covariance matrix of the overall variable *Z* = [*X**Y*] is a block matrix with form

(A.2) $$\Sigma_{Z}=\Sigma_{\left[X,Y\right]}=\left[\begin{array}{cc} \Sigma_{X} & \Sigma_{X;Y}\\ \Sigma_{Y;X} & \Sigma_{Y} \end{array}\right],$$

its determinant can be obtained using the block determinant identity (Horn and Johnson, 2012) as $\left|\Sigma_{\left[X,Y\right]}\right|=\left|\Sigma_{Y}\right|\,\left|\Sigma_{X}-\Sigma_{X;Y}\,\Sigma_{Y}^{-1}\,{\left(\Sigma_{X;Y}\right)}^{T}\right|=\left|\Sigma_{Y}\right|\,\left|\Sigma_{U}\right|$. This last expression leads easily to recover Eq. 5 from the definition of multivariate correlation of Eq. 4.

Finally, we note that both the classic squared correlation and its multivariate extension proposed here have a link with information-theoretic measures when the observed variables have a joint Gaussian distribution. In fact, it is well known that, for scalar Gaussian variables, the variance of *x* is related to the entropy by the equation *H*(*x*) = 0.5*ln*⁡(2π*e*Σ_*x*_), and the partial variance of *x* given *y* is related to the conditional entropy of *x* given *y* by the equation *H*(*x*|*y*) = 0.5*ln*⁡(2π*e*Σ_*x*|*y*_) (Faes et al., 2017a); as a consequence, the squared correlation between *x* and *y* is related to the mutual information by the equation

(A.3) $$I\,\left(x;y\right)=H\,\left(x\right)-H\,\left(x|y\right)=0.5\,\text{ln}\,\frac{\Sigma_{x}}{\Sigma_{x|y}}=-0.5\,\ln\left(1-\rho^{2}\,\left(x;y\right)\right).$$

In the multivariate case when the jointly Gaussian vector variables *X* and *Y* are considered, the relations become *H*(*X*) = 0.5*ln*⁡(^(2π*e*)*P*^|Σ_*X*_|) and *H*(*X*|*Y*) = 0.5*ln*⁡(^(2π*e*)*P*^|Σ_*X*|*Y*_|), which similarly yield

(A.4) $$I\,\left(X;Y\right)=H\,\left(X\right)-H\,\left(X|Y\right)=0.5\,\text{ln}\,\frac{\left|\Sigma_{X}\right|}{\left|\Sigma_{X|Y}\right|}=-0.5\,\ln\left(1-\rho^{2}\,\left(X;Y\right)\right).$$

Therefore, under the hypothesis of joint Gaussianity of *X* and *Y* the MI measure of Eq. 6 is equivalent, up to a factor of two, to the mutual information *I*(*X*;*Y*) between the two variables, i.e., *R*(*X*;*Y*) = 2*I*(*X*;*Y*), and the generalized variances appearing in Eq. 7 are related to the entropy *H*(*X*) and to the conditional entropy of *H*(*X*|*Y*) via the equations *V*(*X*) = 2*H*(*X*)−*P*ln2π*e* and *V*(*X*|*Y*) = 2*H*(*X*|*Y*)−*P*ln2π*e* (Faes et al., 2015c, 2017b). These relations extend to all measures defined in the following in the main text (Eqs 8–11); for instance, in the Gaussian case, the measure of direct interaction between two scalar variables conditioned on all other variables (Eq. 11) takes the form of a conditional mutual information, i.e., *I*(*z*_*i*_;*z*_*j*_|*Z*\{*z*_*i*_,*z*_*j*_}) = *H*(*z*_*j*_|*Z*\{*z*_*i*_,*z*_*j*_})−*H*(*z*_*j*_|*Z*\{*z*_*j*_}) = 0.5*V*(*z*_*j*_|*Z*\{*z*_*i*_,*z*_*j*_})−0.5*V*(*z*_*j*_|*Z*\{*z*_*j*_}) = 0.5*R*(*z*_*i*_;*z*_*j*_|*Z*\{*z*_*i*_,*z*_*j*_}).
