# Supplementary material for: Multivariate Correlation Measures Reveal Structure and Strength of Brain–Body Physiological Networks at Rest and During Mental Stress
Source: Front Neurosci. 2021 Feb 4;14:602584. doi: 10.3389/fnins.2020.602584 (PMC7890264; doi:10.3389/fnins.2020.602584)
Supplement: Supplementary file 1 [file Data_Sheet_1.pdf]

Supplementary Material

Median R(X;Y)			
**Electrode**	**REST**	**MENTAL**	**GAME**
**AF3**	0.054	0.054	0.044
**F7**	0.065	0.059	0.040
**F3**	0.053	0.055	0.057
**FC5**	0.067	0.056	0.042
**T7**	0.055	0.062	0.046
**P7**	0.059	0.071	0.045
**O1**	0.062	0.065	0.052
**O2**	0.054	0.055	0.031
**P8**	0.049	0.058	0.042
**T8**	0.053	0.071	0.048
**FC6**	0.066	0.056	0.046
**F4**	0.068	0.063	0.047
**F8**	0.066	0.065	0.055
**AF4**	0.065	0.042	0.070

F-test R(X;Y) - no. subjects			
**Electrode**	**REST**	**MENTAL**	**GAME**
**AF3**	16	17	12
**F7**	17	16	13
**F3**	16	17	16
**FC5**	17	18	14
**T7**	15	17	16
**P7**	15	17	14
**O1**	17	17	12
**O2**	14	16	9
**P8**	16	16	12
**T8**	17	16	15
**FC6**	17	15	14
**F4**	14	17	15
**F8**	18	17	15
**AF4**	17	16	14

Kruskal-Wallis R(X;Y) - p-value		
**REST**	**MENTAL**	**GAME**
0.417	0.624	0.532

Dunn-Sidak R(X;Y) - p-value			
**Electrode**	**REST-MENTAL**	**REST-GAME**	**MENTAL-GAME**
**AF3**	1.000	0.329	0.289
**F7**	0.965	0.018	0.058
**F3**	0.927	0.981	0.995
**FC5**	0.965	0.124	0.294
**T7**	0.998	0.978	0.934
**P7**	0.767	0.446	0.078
**O1**	0.989	0.130	0.237
**O2**	1.000	0.204	0.175
**P8**	0.948	0.824	0.502
**T8**	0.531	0.858	0.153
**FC6**	0.627	0.076	0.582
**F4**	0.812	0.237	0.734
**F8**	0.858	0.379	0.841
**AF4**	0.033	0.560	0.432

Median $R(x_i;X^i)$			
Process	REST	MENTAL	GAME
η	0.209	0.142	0.085
ϱ	0.256	0.156	0.120
π	0.086	0.048	0.023

F-test $R(x_i;X^i)$ - no. subjects			
Process	REST	MENTAL	GAME
η	18	18	13
ϱ	17	18	15
π	15	11	9

Kruskal-Wallis $R(x_i;X^i)$ - p-value	
Process	p-value
η	0.010
ϱ	0.060
π	0.050

Dunn-Sidak $R(x_i;X^i)$ - p-value			
Process	REST-MENTAL	REST-GAME	MENTAL-GAME
η	0.677	0.010	0.153
ϱ	0.605	0.055	0.517
π	0.121	0.089	0.999

Median $R(y_j;Y^j)$ - REST				
**Electrode**	**δ**	**θ**	**α**	**β**
**AF3**	0.141	0.192	0.134	0.124
**F7**	0.118	0.174	0.136	0.118
**F3**	0.070	0.141	0.157	0.115
**FC5**	0.085	0.166	0.128	0.085
**T7**	0.046	0.076	0.085	0.063
**P7**	0.051	0.123	0.181	0.149
**O1**	0.050	0.105	0.118	0.117
**O2**	0.069	0.119	0.172	0.141
**P8**	0.061	0.126	0.175	0.163
**T8**	0.085	0.144	0.145	0.107
**FC6**	0.088	0.208	0.151	0.113
**F4**	0.074	0.148	0.164	0.134
**F8**	0.152	0.188	0.166	0.120
**AF4**	0.140	0.205	0.165	0.124

Median $R(y_j;Y^j)$ - MENTAL				
**Electrode**	**δ**	**θ**	**α**	**β**
**AF3**	0.321	0.323	0.317	0.156
**F7**	0.242	0.383	0.363	0.184
**F3**	0.092	0.145	0.146	0.092
**FC5**	0.153	0.236	0.164	0.098
**T7**	0.041	0.086	0.138	0.107
**P7**	0.034	0.114	0.113	0.082
**O1**	0.085	0.102	0.145	0.123
**O2**	0.071	0.108	0.133	0.135
**P8**	0.060	0.092	0.107	0.076
**T8**	0.086	0.127	0.110	0.081
**FC6**	0.093	0.124	0.125	0.130
**F4**	0.086	0.137	0.100	0.101
**F8**	0.154	0.248	0.207	0.095
**AF4**	0.288	0.336	0.266	0.086

Median $R(y_j;Y^j)$ - GAME				
**Electrode**	**δ**	**θ**	**α**	**β**
**AF3**	0.100	0.187	0.137	0.118
**F7**	0.113	0.214	0.170	0.132
**F3**	0.068	0.157	0.135	0.113
**FC5**	0.079	0.121	0.112	0.090
**T7**	0.053	0.115	0.102	0.094
**P7**	0.049	0.094	0.072	0.041
**O1**	0.071	0.108	0.129	0.083
**O2**	0.045	0.099	0.092	0.058
**P8**	0.042	0.102	0.111	0.061
**T8**	0.053	0.092	0.105	0.080
**FC6**	0.062	0.154	0.120	0.085
**F4**	0.059	0.134	0.125	0.094
**F8**	0.081	0.176	0.159	0.107
**AF4**	0.081	0.178	0.140	0.101

F-test $R(y_j;Y^j)$ - no. subjects - REST				
**Electrode**	**δ**	**θ**	**α**	**β**
**AF3**	16	18	18	17
**F7**	17	18	18	17
**F3**	18	18	18	16
**FC5**	17	18	18	15
**T7**	14	16	17	16
**P7**	12	17	18	18
**O1**	13	16	17	16
**O2**	14	17	18	18
**P8**	15	16	17	17
**T8**	17	16	17	17
**FC6**	17	18	18	18
**F4**	17	18	18	17
**F8**	17	17	17	17
**AF4**	18	18	18	18

F-test $R(y_j;Y^j)$ - no. subjects - MENTAL				
**Electrode**	**δ**	**θ**	**α**	**β**
**AF3**	16	18	18	17
**F7**	17	18	18	18
**F3**	17	17	17	16
**FC5**	17	18	17	18
**T7**	11	18	16	15
**P7**	9	17	17	16
**O1**	14	17	18	18
**O2**	16	18	18	17
**P8**	15	18	18	17
**T8**	16	18	18	16
**FC6**	15	18	18	16
**F4**	15	18	17	16
**F8**	18	18	17	17
**AF4**	18	18	18	18

F-test $R(y_j;Y^j)$ - no. subjects - GAME				
**Electrode**	**δ**	**θ**	**α**	**β**
**AF3**	15	18	18	17
**F7**	17	18	17	18
**F3**	17	18	18	17
**FC5**	15	18	18	17
**T7**	14	18	16	11
**P7**	13	17	14	10
**O1**	14	18	17	12
**O2**	16	17	16	15
**P8**	14	16	18	14
**T8**	13	17	17	15
**FC6**	15	18	17	16
**F4**	13	18	18	16
**F8**	17	18	18	17
**AF4**	16	18	17	18

Dunn-Sidak $R(y_j;Y^j)$ - p-values - REST-MENTAL				
**Electrode**	**δ**	**θ**	**α**	**β**
**AF3**	0.044	0.010	5.88×10^{-5}	0.252
**F7**	0.080	0.025	0.009	0.247
**F3**	0.335	0.993	0.774	0.987
**FC5**	0.179	0.232	0.734	0.806
**T7**	0.878	0.835	0.774	0.981
**P7**	0.992	1.000	0.196	0.272
**O1**	0.670	0.888	1.000	0.995
**O2**	0.670	1.000	0.852	1.000
**P8**	0.990	0.969	0.582	0.218
**T8**	0.938	0.915	0.670	0.627
**FC6**	0.941	0.538	0.998	0.944
**F4**	0.919	1.000	0.196	0.329
**F8**	1.000	0.204	0.575	0.906
**AF4**	0.085	0.009	0.067	0.780

Dunn-Sidak $R(y_j;Y^j)$ - p-values - REST-GAME				
**Electrode**	**δ**	**θ**	**α**	**β**
**AF3**	0.835	0.976	0.999	1.000
**F7**	0.983	1.000	0.991	0.969
**F3**	0.995	1.000	0.863	0.938
**FC5**	0.996	0.858	1.000	0.911
**T7**	0.998	0.597	0.998	1.000
**P7**	0.974	0.372	0.003	0.001
**O1**	0.734	0.656	0.997	0.379
**O2**	0.605	0.799	0.026	0.019
**P8**	0.481	0.787	0.335	0.026
**T8**	0.294	0.927	0.605	0.793
**FC6**	0.713	0.713	0.289	0.218
**F4**	0.893	0.989	0.495	0.379
**F8**	0.257	0.888	0.987	0.995
**AF4**	0.546	0.974	0.780	1.000

Dunn-Sidak $R(y_j;Y^j)$ - p-values - MENTAL-GAME				
**Electrode**	**δ**	**θ**	**α**	**β**
**AF3**	0.004	0.003	0.000	0.252
**F7**	0.034	0.030	0.004	0.481
**F3**	0.222	0.998	0.998	0.799
**FC5**	0.115	0.044	0.734	0.995
**T7**	0.793	0.976	0.863	0.972
**P7**	0.893	0.353	0.353	0.164
**O1**	1.000	0.972	0.992	0.262
**O2**	0.096	0.787	0.164	0.022
**P8**	0.311	0.962	0.974	0.767
**T8**	0.099	0.590	1.000	0.992
**FC6**	0.959	0.992	0.385	0.502
**F4**	0.538	0.995	0.931	1.000
**F8**	0.262	0.044	0.379	0.974
**AF4**	0.002	0.003	0.005	0.787

Kruskal-Wallis $R(y_j;Y^j)$ - p-values				
**State**	**δ**	**θ**	**α**	**β**
**REST**	8.84×10^{-7}	0.001	0.582	0.461
**MENTAL**	6.43×10^{-12}	2.18×10^{-14}	4.68×10^{-9}	0.057
**GAME**	0.023	0.004	0.167	0.015

Median $R(x_i;Y\|X^i)$ - REST			
Electrode	η	ϱ	π
AF3	0.016	0.018	0.012
F7	0.022	0.019	0.012
F3	0.013	0.015	0.013
FC5	0.022	0.016	0.016
T7	0.017	0.019	0.011
P7	0.022	0.015	0.014
O1	0.018	0.022	0.021
O2	0.023	0.019	0.011
P8	0.020	0.015	0.012
T8	0.022	0.014	0.013
FC6	0.022	0.022	0.013
F4	0.024	0.019	0.015
F8	0.033	0.023	0.014
AF4	0.021	0.018	0.014

Median $R(x_i;Y\|X^i)$ - MENTAL			
Electrode	η	ϱ	π
AF3	0.021	0.027	0.015
F7	0.018	0.025	0.012
F3	0.023	0.019	0.014
FC5	0.021	0.018	0.018
T7	0.020	0.019	0.013
P7	0.023	0.022	0.012
O1	0.022	0.024	0.015
O2	0.023	0.019	0.011
P8	0.023	0.014	0.011
T8	0.033	0.018	0.011
FC6	0.016	0.018	0.013
F4	0.026	0.014	0.013
F8	0.025	0.017	0.015
AF4	0.016	0.016	0.014

Median $R(x_i;Y\|X^i)$ - GAME			
Electrode	η	ϱ	π
AF3	0.015	0.015	0.008
F7	0.014	0.013	0.014
F3	0.020	0.011	0.018
FC5	0.017	0.014	0.013
T7	0.023	0.013	0.013
P7	0.017	0.006	0.013
O1	0.015	0.016	0.009
O2	0.012	0.010	0.009
P8	0.015	0.008	0.012
T8	0.020	0.012	0.017
FC6	0.018	0.013	0.010
F4	0.012	0.011	0.015
F8	0.015	0.020	0.013
AF4	0.015	0.013	0.013

F-test $R(x_i;Y\|X^i)$ - no. subjects - REST			
Electrode	η	ϱ	π
AF3	4	1	1
F7	6	2	0
F3	2	1	0
FC5	5	2	3
T7	4	4	1
P7	4	1	1
O1	5	2	0
O2	5	0	0
P8	2	1	1
T8	5	1	1
FC6	6	3	0
F4	4	3	1
F8	7	3	0
AF4	4	3	1

F-test $R(x_i;Y\|X^i)$ - no. subjects - MENTAL			
Electrode	η	ϱ	π
AF3	3	2	0
F7	5	4	0
F3	4	4	0
FC5	2	3	2
T7	5	4	1
P7	4	4	2
O1	4	4	1
O2	3	0	1
P8	6	4	1
T8	8	1	0
FC6	5	2	0
F4	6	0	0
F8	5	3	1
AF4	2	0	0

F-test $R(x_i;Y\|X^i)$ - no. subjects - GAME			
Electrode	η	ϱ	π
AF3	3	0	1
F7	1	1	1
F3	2	1	1
FC5	3	0	2
T7	5	0	1
P7	1	0	1
O1	2	2	2
O2	1	2	1
P8	2	0	3
T8	2	2	1
FC6	1	1	1
F4	2	1	2
F8	2	4	1
AF4	2	2	3

Dunn-Sidak $R(x_i;Y\|X^i)$ - p-values - REST-MENTAL			
Electrode	η	ϱ	π
AF3	0.858	0.692	0.986
F7	0.888	0.994	0.852
F3	0.411	0.774	0.983
FC5	0.998	0.941	0.976
T7	0.980	0.997	0.980
P7	0.999	0.411	1.000
O1	0.997	0.986	0.835
O2	0.969	0.998	0.830
P8	0.962	0.989	1.000
T8	0.546	0.978	1.000
FC6	0.996	0.967	0.984
F4	0.999	0.727	0.996
F8	0.677	0.754	0.998
AF4	0.418	0.824	0.965

Dunn-Sidak $R(x_i;Y\|X^i)$ - p-values - REST-GAME			
Electrode	η	ϱ	π
AF3	0.806	0.793	0.360
F7	0.078	0.029	0.915
F3	0.474	0.335	0.787
FC5	0.812	0.830	0.962
T7	1.000	0.168	0.999
P7	0.883	0.283	0.931
O1	0.439	0.311	0.109
O2	0.094	0.179	1.000
P8	0.699	0.560	0.906
T8	0.911	0.972	0.793
FC6	0.398	0.283	0.984
F4	0.257	0.200	0.996
F8	0.032	0.754	0.998
AF4	0.385	0.818	0.902

Dunn-Sidak $R(x_i;Y\|X^i)$ - p-values - MENTAL-GAME			
Electrode	η	ϱ	π
AF3	0.341	0.196	0.204
F7	0.311	0.014	0.999
F3	1.000	0.051	0.568
FC5	0.893	0.495	0.806
T7	0.959	0.242	0.995
P7	0.824	0.008	0.893
O1	0.329	0.502	0.459
O2	0.227	0.124	0.774
P8	0.405	0.372	0.919
T8	0.204	0.835	0.754
FC6	0.531	0.538	0.893
F4	0.204	0.767	0.969
F8	0.323	1.000	1.000
AF4	1.000	1.000	0.656

Kruskal-Wallis $R(x_i;Y\|X^i)$ - p-values			
State	η	ϱ	π
REST	0.600	0.490	0.906
MENTAL	0.921	0.639	0.997
GAME	0.752	0.507	0.635

Median $R(y_j;X\|Y^j)$ - REST				
Electrode	δ	θ	α	β
AF3	0.011	0.010	0.012	0.007
F7	0.008	0.018	0.014	0.016
F3	0.013	0.010	0.009	0.009
FC5	0.018	0.010	0.009	0.015
T7	0.019	0.012	0.012	0.009
P7	0.006	0.008	0.010	0.011
O1	0.017	0.007	0.017	0.018
O2	0.013	0.009	0.010	0.013
P8	0.013	0.012	0.007	0.011
T8	0.011	0.009	0.010	0.020
FC6	0.012	0.010	0.012	0.024
F4	0.022	0.012	0.011	0.014
F8	0.015	0.014	0.014	0.022
AF4	0.011	0.013	0.013	0.019

Median $R(y_j;X\|Y^j)$ - MENTAL				
Electrode	δ	θ	α	β
AF3	0.014	0.012	0.005	0.018
F7	0.015	0.007	0.010	0.016
F3	0.009	0.008	0.007	0.017
FC5	0.013	0.006	0.013	0.020
T7	0.015	0.014	0.009	0.013
P7	0.022	0.008	0.012	0.014
O1	0.009	0.011	0.012	0.015
O2	0.012	0.008	0.010	0.012
P8	0.015	0.008	0.010	0.013
T8	0.017	0.007	0.011	0.013
FC6	0.013	0.009	0.007	0.018
F4	0.009	0.007	0.010	0.022
F8	0.012	0.007	0.012	0.018
AF4	0.008	0.009	0.009	0.013

Median $R(y_j;X\|Y^j)$ - GAME				
Electrode	δ	θ	α	β
AF3	0.009	0.008	0.006	0.006
F7	0.008	0.008	0.005	0.014
F3	0.018	0.013	0.012	0.009
FC5	0.013	0.009	0.006	0.012
T7	0.012	0.009	0.018	0.009
P7	0.012	0.007	0.008	0.011
O1	0.013	0.009	0.008	0.012
O2	0.010	0.008	0.005	0.009
P8	0.012	0.009	0.005	0.010
T8	0.015	0.008	0.014	0.007
FC6	0.013	0.008	0.007	0.005
F4	0.011	0.011	0.010	0.009
F8	0.009	0.010	0.010	0.006
AF4	0.009	0.022	0.009	0.007

F-test $R(y_j;X\|Y^j)$ - no. subjects - REST				
Electrode	δ	θ	α	β
AF3	2	0	2	1
F7	2	0	4	2
F3	2	1	1	2
FC5	4	1	3	1
T7	2	2	2	3
P7	1	1	1	3
O1	1	1	3	3
O2	1	1	3	0
P8	2	0	1	2
T8	3	0	0	4
FC6	2	1	0	7
F4	3	0	0	3
F8	1	1	1	7
AF4	2	2	0	3

F-test $R(y_j;X\|Y^j)$ - no. subjects - MENTAL				
Electrode	δ	θ	α	β
AF3	1	2	0	3
F7	4	2	1	5
F3	4	2	0	3
FC5	5	0	0	3
T7	2	2	0	3
P7	4	0	1	3
O1	2	2	4	0
O2	1	0	1	2
P8	3	3	2	3
T8	3	2	0	5
FC6	2	1	2	3
F4	2	1	0	6
F8	1	1	0	3
AF4	0	1	0	2

F-test $R(y_j;X\|Y^j)$ - no. subjects - GAME				
Electrode	δ	θ	α	β
AF3	0	0	2	2
F7	0	0	0	3
F3	5	1	1	2
FC5	1	1	1	2
T7	1	0	1	4
P7	1	0	0	1
O1	1	1	0	2
O2	1	0	0	0
P8	1	1	0	1
T8	4	2	1	0
FC6	0	1	2	3
F4	2	2	2	4
F8	1	2	2	5
AF4	1	4	2	3

Dunn-Sidak $R(y_j;X\|Y^j)$ - p-values - REST-MENTAL				
Electrode	δ	θ	α	β
AF3	0.998	0.799	0.143	0.037
F7	0.994	0.278	0.252	0.911
F3	0.944	0.919	0.787	0.067
FC5	0.906	0.252	0.252	0.677
T7	0.754	0.991	0.747	0.582
P7	0.012	0.969	0.734	0.993
O1	0.677	0.713	0.996	0.793
O2	0.999	0.999	0.995	1.000
P8	0.999	0.670	0.984	0.597
T8	0.761	0.619	0.906	0.931
FC6	0.962	0.897	0.858	0.931
F4	0.509	0.283	0.931	0.459
F8	0.996	0.754	0.883	1.000
AF4	0.873	0.685	0.149	0.379

Dunn-Sidak $R(y_j;X\|Y^j)$ - p-values - REST-GAME				
Electrode	δ	θ	α	β
AF3	0.965	0.976	0.509	0.954
F7	0.824	0.048	0.009	0.993
F3	0.780	0.392	0.873	0.987
FC5	0.453	0.878	1.000	0.923
T7	0.517	0.385	0.706	0.951
P7	0.546	0.806	0.995	0.981
O1	0.806	0.998	0.187	0.793
O2	0.957	0.987	0.032	0.969
P8	0.948	0.670	0.830	0.978
T8	0.873	0.818	0.734	0.392
FC6	0.978	0.998	0.934	0.070
F4	0.272	0.995	0.959	0.741
F8	0.799	0.941	0.685	0.232
AF4	0.998	0.425	0.713	0.063

Dunn-Sidak $R(y_j;X\|Y^j)$ - p-values - MENTAL-GAME				
Electrode	δ	θ	α	β
AF3	0.911	0.553	0.858	0.009
F7	0.677	0.824	0.495	0.787
F3	0.446	0.774	0.341	0.136
FC5	0.847	0.670	0.209	0.311
T7	0.980	0.242	0.175	0.878
P7	0.257	0.969	0.863	0.915
O1	0.996	0.605	0.278	1.000
O2	0.981	0.969	0.057	0.965
P8	0.911	1.000	0.627	0.360
T8	0.996	0.986	0.984	0.139
FC6	1.000	0.957	0.997	0.237
F4	0.972	0.411	1.000	0.074
F8	0.902	0.974	0.981	0.272
AF4	0.780	0.052	0.692	0.780

Kruskal-Wallis $R(y_j;X\|Y^j)$ - p-values				
State	δ	θ	α	β
REST	0.322	0.696	0.762	0.066
MENTAL	0.809	0.666	0.265	0.408
GAME	0.687	0.251	0.048	0.687

Median $R(x_i;X^i,Y)$ - REST			
**Electrode**	**η**	**ϱ**	**π**
**AF3**	0.228	0.282	0.098
**F7**	0.240	0.275	0.104
**F3**	0.234	0.261	0.092
**FC5**	0.241	0.277	0.104
**T7**	0.224	0.265	0.106
**P7**	0.236	0.277	0.104
**O1**	0.221	0.273	0.102
**O2**	0.239	0.263	0.089
**P8**	0.233	0.270	0.096
**T8**	0.218	0.271	0.103
**FC6**	0.245	0.271	0.095
**F4**	0.241	0.284	0.098
**F8**	0.258	0.288	0.095
**AF4**	0.237	0.281	0.096

Median $R(x_i;X^i,Y)$ - MENTAL			
**Electrode**	**η**	**ϱ**	**π**
**AF3**	0.171	0.188	0.066
**F7**	0.177	0.194	0.061
**F3**	0.160	0.175	0.068
**FC5**	0.169	0.177	0.066
**T7**	0.179	0.199	0.068
**P7**	0.179	0.182	0.065
**O1**	0.169	0.187	0.059
**O2**	0.171	0.180	0.063
**P8**	0.192	0.187	0.062
**T8**	0.207	0.179	0.068
**FC6**	0.180	0.191	0.066
**F4**	0.163	0.174	0.064
**F8**	0.176	0.166	0.070
**AF4**	0.157	0.169	0.065

Median $R(x_i;X^i,Y)$ - GAME			
**Electrode**	**η**	**ϱ**	**π**
**AF3**	0.097	0.129	0.046
**F7**	0.092	0.132	0.057
**F3**	0.102	0.131	0.052
**FC5**	0.103	0.133	0.063
**T7**	0.097	0.140	0.051
**P7**	0.093	0.135	0.052
**O1**	0.095	0.139	0.059
**O2**	0.095	0.129	0.052
**P8**	0.098	0.133	0.066
**T8**	0.097	0.130	0.052
**FC6**	0.096	0.131	0.060
**F4**	0.107	0.132	0.056
**F8**	0.102	0.139	0.053
**AF4**	0.102	0.131	0.061

F-test $R(x_i;X^i,Y)$ - no. subjects - REST			
**Electrode**	**η**	**ϱ**	**π**
**AF3**	18	17	13
**F7**	18	18	12
**F3**	18	17	14
**FC5**	18	17	15
**T7**	18	18	15
**P7**	18	18	13
**O1**	18	17	14
**O2**	18	17	12
**P8**	18	18	13
**T8**	18	17	12
**FC6**	18	18	14
**F4**	18	17	15
**F8**	18	17	14
**AF4**	18	17	15

F-test $R(x_i;X^i,Y)$ - no. subjects - MENTAL			
**Electrode**	**η**	**ϱ**	**π**
**AF3**	18	17	10
**F7**	17	17	10
**F3**	18	18	10
**FC5**	17	18	11
**T7**	17	18	12
**P7**	17	17	11
**O1**	18	18	11
**O2**	17	17	10
**P8**	17	17	10
**T8**	17	17	10
**FC6**	17	18	10
**F4**	17	17	11
**F8**	17	18	11
**AF4**	17	18	10

F-test $R(x_i;X^i,Y)$ - no. subjects - GAME			
**Electrode**	**η**	**ϱ**	**π**
**AF3**	14	15	9
**F7**	13	15	10
**F3**	16	15	9
**FC5**	14	15	10
**T7**	16	15	9
**P7**	14	15	10
**O1**	13	16	10
**O2**	13	15	9
**P8**	15	15	10
**T8**	15	15	9
**FC6**	14	15	11
**F4**	13	15	10
**F8**	14	15	10
**AF4**	14	16	11

Dunn-Sidak $R(x_i;X^i,Y)$ - p-values - REST-MENTAL			
**Electrode**	**η**	**ϱ**	**π**
**AF3**	0.787	0.713	0.143
**F7**	0.619	0.663	0.204
**F3**	0.767	0.787	0.085
**FC5**	0.517	0.649	0.130
**T7**	0.677	0.649	0.175
**P7**	0.692	0.663	0.171
**O1**	0.568	0.546	0.187
**O2**	0.685	0.634	0.187
**P8**	0.841	0.767	0.168
**T8**	0.841	0.706	0.171
**FC6**	0.670	0.531	0.213
**F4**	0.641	0.612	0.115
**F8**	0.502	0.605	0.139
**AF4**	0.553	0.531	0.183

Dunn-Sidak $R(x_i;X^i,Y)$ - p-values - REST-GAME			
**Electrode**	**η**	**ϱ**	**π**
**AF3**	0.006	0.055	0.069
**F7**	0.003	0.031	0.289
**F3**	0.018	0.061	0.179
**FC5**	0.002	0.055	0.112
**T7**	0.015	0.043	0.118
**P7**	0.008	0.044	0.183
**O1**	0.005	0.049	0.146
**O2**	0.003	0.045	0.127
**P8**	0.005	0.044	0.289
**T8**	0.006	0.067	0.237
**FC6**	0.005	0.037	0.218
**F4**	0.004	0.087	0.218
**F8**	0.003	0.070	0.096
**AF4**	0.004	0.067	0.300

Dunn-Sidak $R(x_i;X^i,Y)$ - p-values - MENTAL-GAME			
**Electrode**	**η**	**ϱ**	**π**
**AF3**	0.069	0.411	0.986
**F7**	0.080	0.329	0.997
**F3**	0.168	0.366	0.983
**FC5**	0.096	0.474	1.000
**T7**	0.200	0.411	0.997
**P7**	0.121	0.405	1.000
**O1**	0.143	0.546	0.999
**O2**	0.058	0.439	0.997
**P8**	0.049	0.311	0.990
**T8**	0.053	0.467	0.998
**FC6**	0.096	0.488	1.000
**F4**	0.094	0.634	0.987
**F8**	0.124	0.582	0.998
**AF4**	0.127	0.641	0.992

Kruskal-Wallis $R(x_i;X^i,Y)$ - p-values			
**State**	**η**	**ϱ**	**π**
**REST**	1.000	1.000	1.000
**MENTAL**	1.000	1.000	1.000
**GAME**	1.000	1.000	1.000

Median $R(y_j;Y^j|X)$- REST				
Electrode	δ	θ	α	β
AF3	0.153	0.209	0.144	0.135
F7	0.125	0.191	0.148	0.127
F3	0.087	0.144	0.163	0.122
FC5	0.107	0.176	0.141	0.097
T7	0.062	0.087	0.095	0.081
P7	0.059	0.139	0.187	0.170
O1	0.068	0.112	0.152	0.131
O2	0.078	0.123	0.191	0.155
P8	0.075	0.144	0.182	0.181
T8	0.101	0.163	0.154	0.134
FC6	0.110	0.213	0.166	0.177
F4	0.095	0.157	0.169	0.167
F8	0.169	0.201	0.187	0.153
AF4	0.155	0.212	0.177	0.152

Median $R(y_j;Y^j|X)$- MENTAL				
Electrode	δ	θ	α	β
AF3	0.331	0.338	0.319	0.175
F7	0.272	0.391	0.370	0.224
F3	0.112	0.154	0.155	0.125
FC5	0.173	0.238	0.169	0.117
T7	0.049	0.124	0.152	0.127
P7	0.061	0.118	0.129	0.108
O1	0.094	0.114	0.152	0.137
O2	0.084	0.118	0.154	0.150
P8	0.078	0.106	0.134	0.089
T8	0.119	0.148	0.126	0.099
FC6	0.109	0.133	0.144	0.148
F4	0.100	0.147	0.106	0.115
F8	0.183	0.251	0.226	0.132
AF4	0.295	0.346	0.276	0.111

Median $R(y_j;Y^j|X)$- GAME				
Electrode	δ	θ	α	β
AF3	0.111	0.190	0.159	0.126
F7	0.120	0.223	0.176	0.141
F3	0.092	0.159	0.145	0.119
FC5	0.089	0.130	0.130	0.115
T7	0.070	0.126	0.119	0.101
P7	0.071	0.109	0.084	0.061
O1	0.086	0.132	0.140	0.103
O2	0.057	0.114	0.097	0.083
P8	0.059	0.113	0.123	0.074
T8	0.075	0.107	0.107	0.095
FC6	0.072	0.172	0.135	0.087
F4	0.083	0.147	0.144	0.098
F8	0.096	0.185	0.168	0.145
AF4	0.105	0.191	0.162	0.126

F-test $R(y_j;Y^j|X)$ - no. subjects - REST				
Electrode	δ	θ	α	β
AF3	15	18	18	16
F7	17	18	18	17
F3	16	18	18	15
FC5	16	18	15	15
T7	14	15	17	16
P7	10	16	18	18
O1	11	16	17	16
O2	13	17	18	17
P8	16	16	16	16
T8	16	15	17	15
FC6	16	18	17	17
F4	16	17	18	17
F8	17	17	17	17
AF4	16	18	18	18

F-test $R(y_j;Y^j|X)$ - no. subjects - MENTAL				
Electrode	δ	θ	α	β
AF3	16	18	18	17
F7	17	18	18	17
F3	17	18	15	15
FC5	17	18	17	17
T7	9	16	16	14
P7	10	17	15	15
O1	13	16	17	17
O2	17	18	18	18
P8	14	17	16	17
T8	16	18	15	16
FC6	14	18	17	17
F4	16	17	16	17
F8	17	18	17	18
AF4	18	18	18	18

F-test $R(y_j;Y^j|X)$ - no. subjects - GAME				
Electrode	δ	θ	α	β
AF3	14	18	17	15
F7	15	18	17	17
F3	15	18	17	14
FC5	14	17	18	17
T7	13	16	15	11
P7	12	16	13	11
O1	12	18	17	13
O2	11	16	16	12
P8	12	15	16	14
T8	12	16	15	13
FC6	14	17	16	13
F4	11	17	17	14
F8	15	18	17	16
AF4	15	18	17	16

Dunn-Sidak $R(y_j;Y^j|X)$ - p-values - REST-MENTAL				
Electrode	δ	θ	α	β
AF3	0.063	0.007	6.78×10^{-5}	0.149
F7	0.087	0.033	0.014	0.164
F3	0.553	0.981	0.799	0.986
FC5	0.218	0.257	0.835	0.720
T7	0.754	0.824	0.841	0.847
P7	0.799	0.998	0.262	0.267
O1	0.799	0.627	1.000	0.998
O2	0.754	1.000	0.767	1.000
P8	0.987	0.944	0.699	0.317
T8	0.774	0.897	0.818	0.627
FC6	0.911	0.560	0.998	0.944
F4	0.994	1.000	0.242	0.495
F8	0.998	0.289	0.663	0.852
AF4	0.099	0.012	0.076	0.888

Dunn-Sidak $R(y_j;Y^j|X)$ - p-values - REST-GAME				
Electrode	δ	θ	α	β
AF3	0.727	0.957	1.000	0.998
F7	0.948	0.999	0.965	0.972
F3	0.999	0.999	0.915	0.873
FC5	0.938	0.883	0.995	0.927
T7	1.000	0.741	0.954	1.000
P7	0.858	0.247	0.003	0.001
O1	0.873	0.411	0.906	0.360
O2	0.453	0.727	0.012	0.018
P8	0.405	0.824	0.329	0.025
T8	0.432	0.911	0.747	0.560
FC6	0.575	0.713	0.335	0.094
F4	0.641	0.992	0.531	0.283
F8	0.300	0.897	0.972	0.938
AF4	0.656	0.993	0.774	1.000

Dunn-Sidak $R(y_j;Y^j|X)$ - p-values - MENTAL-GAME				
Electrode	δ	θ	α	β
AF3	0.004	0.001	5.61×10^{-5}	0.209
F7	0.023	0.023	0.003	0.347
F3	0.634	0.994	0.994	0.976
FC5	0.067	0.058	0.706	0.972
T7	0.713	0.999	0.990	0.830
P7	0.999	0.323	0.278	0.160
O1	0.999	0.984	0.878	0.278
O2	0.076	0.720	0.127	0.016
P8	0.242	0.990	0.923	0.627
T8	0.076	0.531	0.999	1.000
FC6	0.923	0.995	0.432	0.272
F4	0.481	0.998	0.948	0.980
F8	0.222	0.074	0.398	0.996
AF4	0.005	0.005	0.006	0.923

Kruskal-Wallis $R(y_j;Y^j|X)$ - p-values				
State	δ	θ	α	β
REST	7.13×10^{-6}	3.28×10^{-4}	0.619	0.328
MENTAL	6.87×10^{-11}	6.42×10^{-15}	9.08×10^{-9}	0.061
GAME	0.054	0.002	0.138	0.030
